# Salmonella enterica Serovar Typhimurium Uses PbgA/YejM To Regulate Lipopolysaccharide Assembly during Bacteremia

**DOI:** 10.1128/IAI.00758-19

**Published:** 2019-12-17

**Authors:** Melina B. Cian, Nicole P. Giordano, Revathi Masilamani, Keaton E. Minor, Zachary D. Dalebroux

**Affiliations:** a Department of Microbiology and Immunology, University of Oklahoma Health Sciences Center, Oklahoma City, Oklahoma, USA; University of California San Diego School of Medicine

**Keywords:** outer membrane, fatty acid, cyclopropane, cardiolipin, Toll-like receptor 4, unsaturated, saturated, phosphatidylglycerol, phosphatidylethanolamine, lipooligosaccharide, O antigen, core oligosaccharide, O polysaccharide, LPS precursor, lipid A, endotoxin, LapB/YciM, FtsH, LpxC, PhoQ, PhoP, two-component system, persistence, intracellular pathogen, macrophage, rifampin, lipid A-core, nontyphoidal, lipidomics, capsule, RcsF, colonic acid, *Salmonella*, capsular polysaccharide, facultatively intracellular pathogens, fatty acids, lipopolysaccharide, glycerophospholipids, phospholipids

## Abstract

Salmonella enterica serovar Typhimurium (*S*. Typhimurium) relies upon the inner membrane protein PbgA to enhance outer membrane (OM) integrity and promote virulence in mice. The PbgA transmembrane domain (residues 1 to 190) is essential for viability, while the periplasmic domain (residues 191 to 586) is dispensable. Residues within the basic region (residues 191 to 245) bind acidic phosphates on polar phospholipids, like for cardiolipins, and are necessary for salmonella OM integrity. *S*. Typhimurium bacteria increase their OM cardiolipin concentrations during activation of the PhoPQ regulators. The mechanism involves PbgA’s periplasmic globular region (residues 245 to 586), but the biological role of increasing cardiolipins on the surface is not understood. Nonsynonymous polymorphisms in three essential lipopolysaccharide (LPS) synthesis regulators, *lapB* (also known as *yciM*), *ftsH*, and *lpxC*, variably suppressed the defects in OM integrity, rifampin resistance, survival in macrophages, and systemic colonization of mice in the *pbgA*Δ*191–586* mutant (in which the PbgA periplasmic domain from residues 191 to 586 is deleted). Compared to the OMs of the wild-type salmonellae, the OMs of the *pbgA* mutants had increased levels of lipid A-core molecules, cardiolipins, and phosphatidylethanolamines and decreased levels of specific phospholipids with cyclopropanated fatty acids. Complementation and substitution mutations in LapB and LpxC generally restored the phospholipid and LPS assembly defects for the *pbgA* mutants. During bacteremia, mice infected with the *pbgA* mutants survived and cleared the bacteria, while animals infected with wild-type salmonellae succumbed within 1 week. Remarkably, wild-type mice survived asymptomatically with *pbgA-lpxC* salmonellae in their livers and spleens for months, but Toll-like receptor 4-deficient animals succumbed to these infections within roughly 1 week. In summary, *S*. Typhimurium uses PbgA to influence LPS assembly during stress in order to survive, adapt, and proliferate within the host environment.

## Abstract (precis)

Salmonella enterica serovar Typhimurium (*S*. Typhimurium) relies upon the inner membrane protein PbgA to enhance outer membrane (OM) integrity and promote virulence in mice. The PbgA transmembrane domain (residues 1 to 190) is essential for viability, while the periplasmic domain (residues 191 to 586) is dispensable. Residues within the basic region (residues 191 to 245) bind acidic phosphates on polar phospholipids, like for cardiolipins, and are necessary for salmonella OM integrity. *S*.

## INTRODUCTION

Salmonella enterica serovar Typhimurium (*S*. Typhimurium) is a Gram-negative facultative intracellular pathogen that causes gastroenteritis in healthy humans and bacteremia in immunocompromised individuals (1). During systemic pathogenesis, *S*. Typhimurium resists several host-killing strategies, including degradation within the phagolysosome vacuoles of macrophages (2, 3). Vacuolar salmonellae sense the acidic pH, low levels of divalent cations, and high levels of antimicrobial peptides within the phagolysosome and respond by remodeling the phospholipid and lipopolysaccharide (LPS) constituents for their outer membrane (OM) bilayer (2, 4, 5). Although several *S*. Typhimurium lipid-remodeling mechanisms exist, those critical for survival during bacteremia are largely unknown.

**FIG 1:**
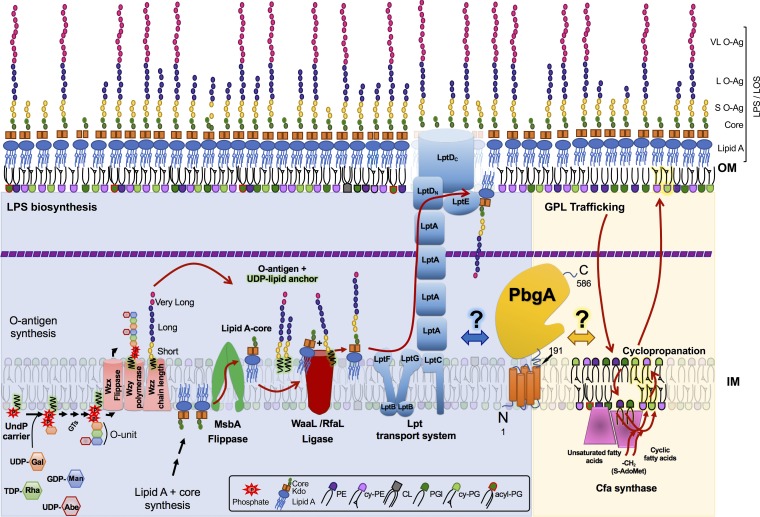
Model of lipopolysaccharide (LPS) synthesis and assembly pathways for Gram-negative bacteria and a schematic representing the predicted functional role(s) of PbgA/YejM in phospholipid trafficking, remodeling, and homeostasis. The left side of the model shows the LPS biogenesis pathway for Salmonella enterica (blue background). Briefly, LPS is synthesized from two precursors, lipid A-core molecules and O antigens, by two pathways. LPS assembly involves the ligation of O antigens to lipid A-core molecules and results in the formation of short, long, and very long LPS modalities for *S*. Typhimurium. O-antigen synthesis begins at the inner leaflet of the inner membrane (IM) by a sequential transfer of monosaccharides from sugar-nucleotide donor molecules to the undecaprenyl phosphate carrier (Und-PP carrier or C55-PP) lipid to form a Und-PP-linked unit. In *S*. Typhimurium, the repeating unit is a four-residue branched structure (galactose [Gal], rhamnose [Rha], mannose [Man], abequose [Abe]) synthesized by the glycosyltransferases (GTs) WbaP, WbaN, WbaU, and WbaV, respectively. Lipid A-core biosynthesis begins in the cytoplasm and continues in parallel with O-antigen synthesis upon the IM inner leaflet, evoking nine conserved enzymes of the Raetz pathway. Two 3-deoxy-d-manno-oct-2-ulosonic acid (Kdo) residues are added to serve as acceptors for the sugar groups composing the core oligosaccharide. The flippase MsbA inverts lipid A-core molecules into the periplasmic leaflet of the IM. Next, the O-antigen and lipid A-core structures are joined with WaaL also known as RfaL into one LPS superstructure. The Lpt complex spans the dual bilayers of the envelope and drives unidirectional LPS transport across the periplasm. The right side of the model depicts the predicted PbgA also known as YejM involvement in the phospholipid trafficking pathways necessary for *Enterobacteriaceae* to catalyze cyclopropane ring formation on phospholipids during stress (yellow background). Cyclopropanated fatty acids (Cfa) are formed by the addition of a methylene group from *S*-adenosylmethionine (S-AdoMet) across the carbon-carbon double bond of unsaturated fatty acids. This takes place at the cytosolic surface of the IM and is catalyzed by Cfa, which functions as a dimer. For bacteria to remodel unsaturated fatty acids on the phospholipids that exist within the inner leaflet of the OM at the time of stress, they must traffic the molecules back to the IM and somehow invert them into the cytosolic leaflet. Many protein systems involved in these trafficking mechanisms remain undiscovered, but the Mla system is the most well studied example of an intermembrane bacterial phospholipid transport system. VL O-Ag, L O-Ag, and S O-Ag, very long, long, and short O antigens, respectively; LOS, lipooligosaccharide.

The cell envelope of the *Enterobacteriaceae* consists of two concentric membranes separated by a periplasmic space and a thin layer of peptidoglycan (Fig. 1). The inner membrane (IM) is symmetric, encases the cytosol, and consists of phospholipids. The OM is asymmetric, surrounds the periplasm, and consists of inner leaflet phospholipids and outer leaflet LPS molecules (Fig. 1) (6, 7). Peptidoglycan is attached to the OM by lipoproteins and imparts cell shape to the bacterium. OM lipoproteins are typically anchored to phospholipids, but some can adopt transmembrane configurations and access the LPS molecules within the outer leaflet (8). Lipid asymmetry and LPS biochemistry provide chemical-physical barrier properties to the OM that are critical for Gram-negative bacteria to resist antibiotics and withstand immune systems (9, 10).

Lipid A molecules are multiply acylated disaccharolipids that form the amphipathic base structure of LPS and bind underlying phospholipids (11). In addition to promoting the OM barrier, lipid A molecules are potent endotoxins and microbe-associated molecular patterns that bind and activate the mammalian Toll-like receptor 4 (TLR4) complex and the noncanonical inflammasome receptor complexes: mouse caspase-11 and human caspase-4 and -5 (12, 13). Receptor activation prompts proinflammatory cytokine production and eukaryotic cell death (14, 15). Pathogens, including *S*. Typhimurium, regulate the chemical structure, overall level, and extracellular release of LPS molecules to enhance their fitness in their hosts (5).

The LPS glycolipids consist of the lipid A disaccharolipid, the core oligosaccharide, and the O polysaccharide. Lipid A is built in the cytosol and upon the IM by the essential Lpx enzymes (16) (Fig. 1). Inner and outer core oligosaccharides are constructed in the cytosol and attached to lipid A molecules at the IM, and this completes the lipid A-core assembly (17). The essential flippase MsbA inverts lipid A-core glycolipids into the periplasmic leaflet of the IM (18). In parallel, *Enterobacteriaceae* build O-antigen subunits in the cytosol and transfer them to an undecaprenyl phosphate Und-PP carrier lipid to link them to the IM (17). The flippase Wzx flips UDP-O antigens into the periplasmic leaflet of the IM, where the Wzy-Wzz complex polymerizes the subunits into multiple modalities of various chain lengths (Fig. 1) (17). The O-antigen ligase WaaL then nonselectively ligates the polymers to lipid A-core structures, and the Lpt system rapidly transports the fully assembled LPS molecules to the OM and inserts them into the outer leaflet (Fig. 1) (19–21). *S*. Typhimurium displays a trimodal arrangement of LPS structures on the cell surface, which consist of short (2 to 15 repeating units [RU]), long (16 to 35 RU), or very long (>100 RU) O-antigen subtypes (4). *S*. Typhimurium regulates the relative abundance of the three LPS modalities on the surface, and the O-antigen subunits are critical for the OM barrier function, antibiotic resistance, and virulence (22–28).

*Enterobacteriaceae* produce four major phospholipid families, including the phosphatidylethanolamines (PEs), the phosphatidylglycerols (PGls), the cardiolipins (CLs), and the acyl-phosphatidylglycerols (aPGls), which constitute roughly 70, 20, 5, and 2% of the total membrane phospholipid composition for *S*. Typhimurium, respectively (29–31). Phospholipids typically harbor one, two, three, or four fatty acids that are either saturated or monounsaturated and that have carbon lengths of 14, 16, or 18 atoms (32, 33). During stress, *Enterobacteriaceae* downregulate phospholipid biosynthesis and upregulate cyclopropane fatty acid synthase (Cfa) (34). Cfa is an induced cytosolic enzyme that binds the IM, dimerizes, and transfers a methylene group from *S*-adenosylmethionine to monounsaturated fatty acids on existing phospholipids (Fig. 1) (35, 36). Bacteria cyclopropanate nearly all the unsaturated fatty acids on existing phospholipids within the envelope during stationary-phase stress (34, 37–39). This requires them to traffic OM phospholipids back to the inner leaflet of the IM to be accessed by Cfa dimers (Fig. 1). Unlike LPS transport, phospholipid transport is bidirectional, and several systems exist; however, only Mla has been shown to mediate a direct transfer mechanism (40–46).

Gram-negative bacteria generally maintain OM-lipid asymmetry using the MlaA/VacJ-OmpC complex and the OM phospholipase (OMPLA) (47). Intriguingly, *S*. Typhimurium constitutively inverts phospholipids into the OM outer leaflet as a function of the PhoPQ two-component virulence regulators, which are activated in macrophage phagolysosomes and increase the expression of genes that biochemically remodel the structure of LPS molecules (4, 48). Outer leaflet phospholipids are substrates for the PhoPQ-activated gene product PagP, an OM phospholipase A1/palmitoyltransferase enzyme that transfers palmitoyl groups to PGls and LPS molecules within the OM outer leaflet (4). Heptaacylated lipid A structures elicit reduced TLR4 activation compared to hexaacylated precursors and promote resistance to helical antimicrobial peptides, but the role of the triacylated aPGls is not known (5, 49). Therefore, *S*. Typhimurium regulates phospholipid flipping into the outer leaflet of the OM during infection to alter the properties of LPS molecules.

We showed that *S*. Typhimurium uses PbgA/YejM, a conserved enterobacterial IM protein, to enhance OM integrity and mediate survival in macrophages and mice (Fig. 1) (50). The transmembrane domain of PbgA (residues 1 to 190) consists of five helices that are essential for enterobacterial viability, while the periplasmic domain (residues 191 to 586) is dispensable (50–54). The periplasmic domain consists of the basic region (residues 191 to 245) and the globular region (residues 245 to 586). The basic region binds phospholipids and has a greater affinity for CLs than for PGls or PEs (50). Consecutive arginines (R215, R216) within the basic region bind anionic lipid head groups and are necessary for the *S*. Typhimurium OM barrier function (50). The PbgA globular region is required for *S*. Typhimurium to increase the CL content of the OM during activation of the PhoPQ regulators, but PhoPQ does not control PbgA levels (50). Further, deleting the PbgA globular region does not impact OM CL levels when PhoPQ are not activated. Finally, CL phospholipids are dispensable for enterobacterial viability, while PbgA transmembrane segments are essential (50–54). Therefore, we pursued the hypothesis that the periplasmic domain of PbgA contributes to a central mechanism of *S*. Typhimurium lipid homeostasis.

## RESULTS

### *S*. Typhimurium PbgA/YejM promotes LPS integrity during stress.

The OM sensor lipoprotein RcsF adopts surface-exposed transmembrane configurations, which allows *Enterobacteriaceae* to detect LPS integrity within the outer leaflet (55, 56). Once activated, RcsF initiates a phosphorelay at the IM that activates the *wza* operon, which encodes the synthesis and export proteins for the production of the colonic acid exopolysaccharide capsule (57). Our lab routinely monitors a chromosomally integrated *wza-lacZ* gene reporter to quantify bacterial defects in OM lipid integrity (58). *S*. Typhimurium mutants in which the PbgA periplasmic domain from residues 191 to 586 is deleted, referred to as *pbgA*Δ*191–586* mutants, have increased RcsF activity and permeation across the OM under conditions when PhoPQ are not active (50, 58). Therefore, we hypothesized that *S*. Typhimurium uses PbgA to promote LPS integrity.

**FIG 2:**
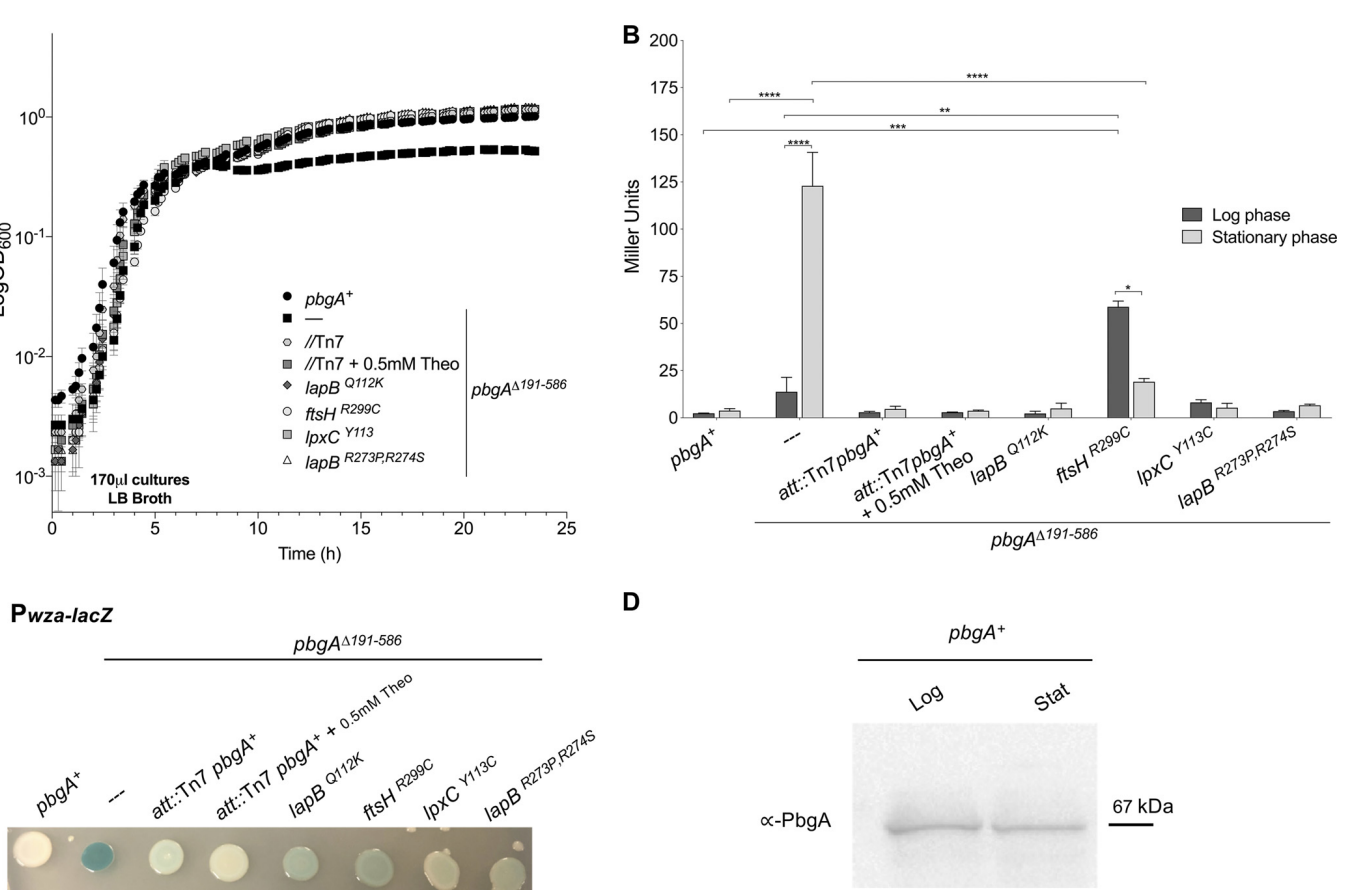
Salmonella enterica serovar Typhimurium (*S*. Typhimurium) requires the periplasmic domain of PbgA to promote the growth transition to stationary phase and outer membrane (OM) integrity. (A) Bacterial growth in small-volume cultures (170 μl) was monitored using a multiwell plate and a growth curve analyzer. Cultures were initiated from single-colony dilutions. Density measurements were made every 0.5 h for 24 h. Data reflect the mean ± SD for triplicate wells and two experiments. (B) β-Galactosidase activity was quantified for bacteria cultured in Luria-Bertani (LB) broth to either the log phase (optical density at 600 nm [OD_600_], 0.6 to 0.8) or the stationary phase (16 h after single-colony inoculation; see Materials and Methods). Average values (Miller units) ± standard error of the mean (SEM) were calculated. Two-way analysis of variance followed by Sidak’s multicomparison test was done for statistical analyses. *, *P <* 0.05; **, *P* < 0.01; ***, *P *< 0.001; ****, *P* < 0.0001; no asterisk indicates not significant. (C) Each *S*. Typhimurium genotype used in this study harbors the *wza-lacZ* gene reporter of Rcs signaling activity. The broth-grown wild-type (*pbgA^+^*) strain, the *pbgA* periplasmic domain deletion mutant (the *pbgA*Δ*191–586* mutant), the strain with the complementation genotype (the *pbgA*Δ*191–586 att*::Tn*7-pbgA^+^* [//Tn7] strain), and the four suppressor isolates were plated onto LB agar containing the β-galactosidase (LacZ) indicator substrate X-Gal (5-bromo-4-chloro-3-indolyl-β-d-galactopyranoside; 20 μg/ml) (Table 1). The *pbgA*Δ*191–586 att*::Tn*7-pbgA^+^* strain was cultured in broth with or without 0.5 mM theophylline (Theo); theophylline was used to achieve either basal or PbgA overexpression (see Fig. S3 in the supplemental material). (D) A Western blot in which an antibody directed to the PbgA periplasmic domain was used. The total membrane fractions of the log-phase (Log) and stationary-phase (Stat) wild-type *S*. Typhimurium bacteria were probed for PbgA abundance.

To define the mechanism, we cultured wild-type and *pbgA*Δ*191–586* mutant *S*. Typhimurium in a rich broth medium, monitored the bacterial growth rate, and quantified the levels of the *wza-lacZ* reporter (Fig. 2A to C; see Table S1 and Fig. S1 and S2 in the supplemental material). During the log phase of growth, the *pbgA* mutants replicated at rates that were comparable to those for the wild type, but heightened *wza-lacZ* reporter activity was measured (Fig. 2A to C; Fig. S1 and S2). Near the stationary phase, the *pbgA* mutants prematurely stalled their growth rates compared to the rate for the wild type and increased their gene reporter levels above that of the log phase (Fig. 2A and B; Fig. S1 and S2). Whole-cell extracts for wild-type (*pbgA^+^*) *S*. Typhimurium revealed no obvious variation in PbgA abundance between the growth phases (Fig. 2D). Therefore, *S*. Typhimurium uses PbgA to promote LPS integrity during stress without regulating PbgA levels.

To complement the mutant phenotypes, *pbgA* was cloned downstream of a constitutive promoter and a synthetic riboswitch and inserted onto the *pbgA*Δ*191–586* mutant chromosome at a neutral locus (59, 60). The riboswitch was otherwise absent from the genome and works by repressing translation until theophylline, a small molecule that acts as an inducer and that is not toxic or a carbon source, is added to the medium (58, 59). Without theophylline, the bacteria with the rescue genotype expressed low levels of PbgA that were similar to those expressed by the wild type (Fig. S3). Low-level PbgA expression was fully sufficient to restore the growth and LPS integrity defect for the *pbgA* mutants (Fig. 2A to C; Fig. S3). Adding theophylline caused PbgA overexpression and also rescued the mutant defects (Fig. 2A to C; Fig. S1). Therefore, the phenotypes were largely dependent upon the loss of PbgA and not on some other genetic perturbation.

### *S*. Typhimurium requires the PbgA periplasmic domain to colonize mice, and *pbgA* mutant suppressor variants emerge from the host environment with restored LPS integrity.

**FIG 3:**
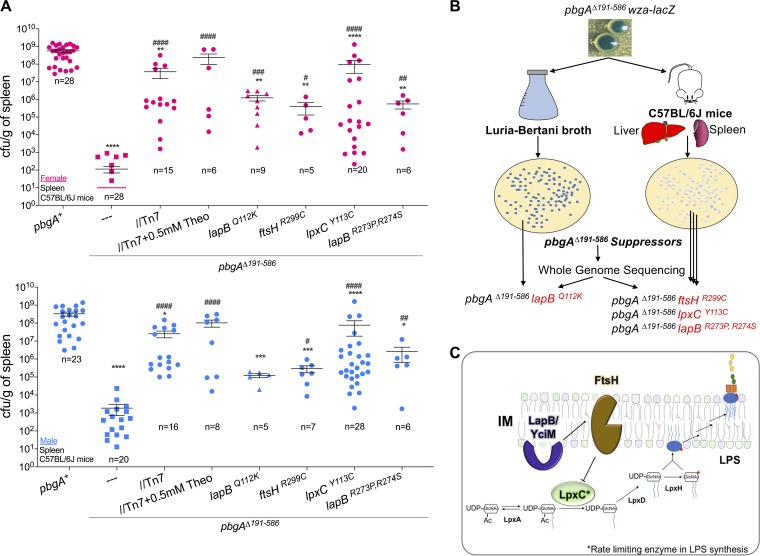
The PbgA periplasmic domain is necessary for systemic pathogenesis in mice, and only spontaneous suppressors were recovered from mice infected with *pbgA* mutants. (A) Female (top) and male (bottom) C57BL/6J mice were intraperitoneally (i.p.) injected with ∼5 × 10^5^ CFU of the wild type, the *pbgA* mutant, the complemented mutant, or the *pbgA* mutant suppressor variants (Table 1). After 2 days postinfection (pi), the mice were euthanized and colony counts were enumerated from spleen homogenates. Data are shown as the mean number of CFU per gram of spleen ± SEM. Each genotype was assessed in at least five mice (*n* = 5). Some strains were tested in a higher number of mice to ensure accuracy. The limit of detection was 10 CFU for the spleen. A one-way analysis of variance and the Kruskal-Wallis multicomparison test were done, which allowed us to compare groups of different sizes, given the nonnormal distribution of the data. Asterisks or number symbols indicate a statistically significant difference relative to the results for the wild type or the *pbgA* mutant, respectively. *, *P* < 0.05; **, *P* < 0.01; ***, *P* < 0.001; ****, *P* < 0.0001; no asterisk indicates not significant. (B) Schematic detailing our genetic screen to isolate spontaneous suppressors of the *pbgA* mutant LPS integrity defect. Extended culturing in stationary phase and exposure to the systemic host environment of mice enrich for *pbgA* mutant suppressor variants that are light blue or white on indicator agar. The genomes of one suppressor isolated from a broth culture and three suppressors isolated from the livers and spleens of male mice were sequenced alongside those of isolates with the wild-type and the *pbgA* mutant parental genotypes to define the nonsynonymous single-nucleotide polymorphisms (SNPs) that are listed (Table 1). (C) SNPs were found in key genes that regulate lipid A-core biosynthesis. The LapB also known as YciM protein is induced in stationary phase and in response to heat stress and functions as a negative regulator of LPS levels. This occurs since LapB binds FtsH, a pleiotropic protease, and activates FtsH digestion of LpxC, the rate-limiting enzyme in the lipid A biosynthesis pathway.

As part of understanding the biochemical role of PbgA in promoting LPS integrity, we sought to quantify the contribution of the periplasmic domain to *S*. Typhimurium colonization and survival in mice. Male and female C57BL/6J animals were intraperitoneally (i.p.) injected with roughly 10^5^ salmonellae encoding the *wza-lacZ* reporter. By 48 h, wild-type *S*. Typhimurium achieved titers of between 10^7^ and 10^9^ CFU per gram of organ tissue in the spleens and livers of the mice (Fig. 3A; Fig. S4). In contrast, the *pbgA* mutants were largely decimated by mice and achieved titers of only between 10^1^ and 10^4^ CFU/g at this time point (Fig. 3A; Fig. S4). Females were more resistant than males to *pbgA* mutant colonization, since only 25% of the females but 100% of the males became measurably colonized by 2 days (Fig. 3A; Fig. S4). The complemented mutants achieved average numbers of CFU per gram that were several logs greater than those of the deletion mutants in mice of both genders; however, the titers were still significantly less than those of the wild type (Fig. 3A; Fig. S4). Therefore, second-site PbgA expression was not fully sufficient to rescue the mutant defect under these conditions.

The *pbgA*Δ*191–586* mutant colonies that emerged from mice were always light blue or white on indicator agar, suggesting that their LPS integrity defect had been restored (Fig. 2C and 3A; Fig. S4). The possibility that the host environment enriched for *pbgA* mutant suppressor variants inspired us to sequence the genomes of the individual isolates (Table 1; Fig. 2C and 3B; Fig. S4). We reasoned that by identifying the causative genetic changes we could better understand the biological function of PbgA for *S*. Typhimurium.

**TABLE 1:** Whole-genome sequencing results for the suppressor isolates which emerged from the Salmonella enterica serovar Typhimurium strain with the *pbgA*Δ*191–586 wza-lacZ* genotype^*a*^

Isolate	Origin of isolate	Color after plating on X-Gal	Reference base(s)	Variant base(s)	Locus	Gene name	Protein product	Peptide change(s)	No. of reads	Frequency
Suppressor A	Male mouse liver	Light blue	G	A	STM14_3981	*ftsH* (*hflB*)	ATP-dependent zinc metalloprotease that degrades LpxC	299R → C	117	1.00
Suppressor B	Male mouse spleen	White	C, G	G, T	STM14_2066	*lapB* (*yciM*)	Tetratricopeptide repeat protein, negative LpxC regulator	273R → P, 274R → S	156	0.99, 1.00
Suppressor C	Male mouse liver	White	A	G	STM14_0160	*lpxC*	UDP-3-*O*-acyl *N*-acetylglucosamine deacetylase	113Y → C	98	0.99
Suppressor D	Overnight culture on LB broth	White	G	T	STM14_2066	*lapB* (*yciM*)	Tetratricopeptide repeat protein, negative LpxC regulator	112Q → K	137	1.00

a All isolates had nonsynonymous polymorphisms. X-Gal, 5-bromo-4-chloro-3-indolyl-β-d-galactopyranoside.

### Single-nucleotide polymorphisms (SNPs) in *lapB*, *ftsH*, and *lpxC* restore the LPS integrity defect for *pbgA* mutants.

Culturing *pbgA* mutants to late stationary phase in nutrient-rich broth medium also yielded white colony suppressor variants, albeit at a lower frequency compared with the occurrence in mice (Fig. 3B). The genomes of four suppressors were sequenced; three were from infected male mice, and one was from a stationary-phase culture (Table 1). Using the wild-type and *pbgA*Δ*191–586* mutant genomes as references, we performed an analysis for nucleotide variants and identified five distinct nonsynonymous polymorphisms (Table 1). In particular, substitution mutations were mapped to three essential proteins that are known regulators of enterobacterial LPS biosynthesis, LapB also known as YciM, FtsH, and LpxC (Fig. 3C) (61).

The genome of the suppressor from the stationary-phase culture encoded a single-amino-acid substitution in LapB, Q112K (Table 1). The three suppressors from mice encoded a single substitution in LpxC (Y113C), a single substitution in FtsH (R299C), and two consecutive substitutions in a pair of arginines for LapB (R273P, R274S) (Table 1; Fig. 3B and C). LpxC is the cytosolic deacetylase that controls a rate-limiting step for lipid A biosynthesis (16). FtsH is the IM protease that degrades LpxC and other key proteins involved in phospholipid and LPS biosynthesis (61, 62). LapB is an essential tetratricopeptide repeat protein that is anchored to the IM and localized to the cytosol. LapB binds LPS molecules, as well as proteins that synthesize, assemble, and transport LPS molecules (63, 64). Escherichia coli increases its LapB levels during stress. LapB binds FtsH and relays unknown fatty acid signals that prompt FtsH to degrade LpxC and likely other proteins (Fig. 3C) (63–66). To summarize, the genetics supported the suggestion that *S*. Typhimurium requires PbgA to regulate LPS biosynthesis.

### *S*. Typhimurium uses PbgA to promote LPS assembly.

**FIG 4:**
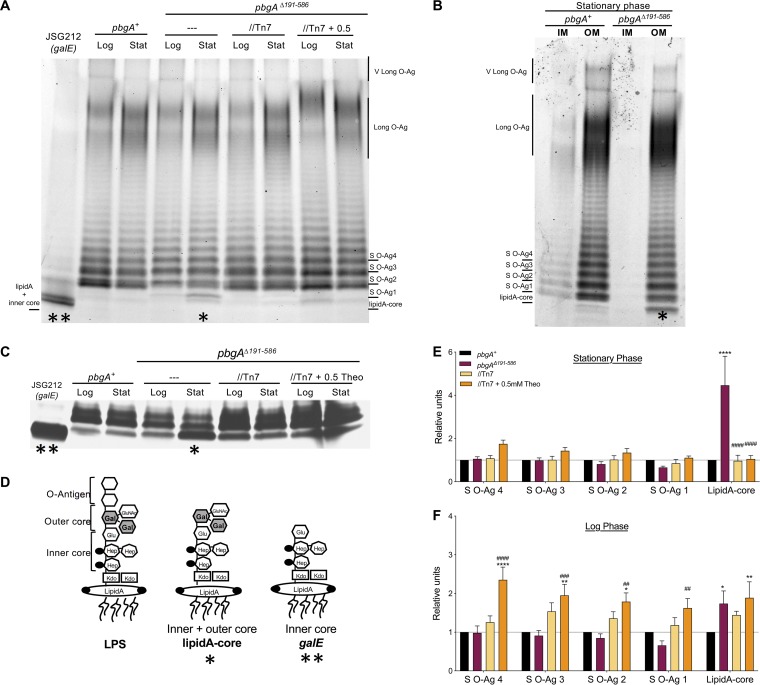
PbgA impacts LPS assembly in a growth phase-dependent manner. (A) Hot phenol extraction of LPS was performed using log- and stationary-phase cultures of the wild-type salmonellae, the *pbgA* mutant, and the strain with the complemented mutant genotype after normalization to an optical density at 600 nm of 2.0. The *S*. Typhimurium Δ*galE* mutant was used as a control since it produces a truncated lipid A inner core yet is devoid of the outer core oligosaccharide and the O antigen. LPS was extracted, electrophoresed, and stained with ProQ300 Emerald. The gel represents that from one of five independent experiments. *, lipid A-core; **, lipid A-inner core. (B) Inner membrane (IM) and outer membrane (OM) fractions were isolated from stationary-phase cultures of the wild type (*pbgA^+^*) and the *pbgA* mutant. Samples were normalized based on the total protein concentration, and LPS was extracted, electrophoresed, and stained with ProQ300 Emerald. The gel represents that from one of two experiments. (C) The ProQ300 Emerald-stained gels were costained with silver to better visualize the lower-molecular-weight lipid A-core bands. *, lipid A-core; **, lipid A-inner core. (D) Depiction of lipopolysaccharide (LPS) structural domains: lipid A, the core oligosaccharide (inner and outer), and the O antigen. The core structure contains 3-deoxy-d-manno-oct-2-ulosonic acid (Kdo) residues, heptoses, glucoses, and galactoses. The Δ*galE* mutant produces a partial molecule, comprised of the lipid A inner core region. (E and F) The relative band intensity compared to that of the wild type was measured for four selected short-length O-antigen (S O-Ag) molecules and the predominant lipid A-core intermediate from *S*. Typhimurium. Band intensities, determined using Image Lab software (Bio-Rad), were obtained from ProQ300 Emerald-stained gels for the *pbgA^+^* strain, the *pbgA* mutant, and the strain with the complementation genotype cultured to the stationary phase (E) or the log phase (F). Each band for the *pbgA* mutant derivatives was normalized to the band for the wild type in that particular growth phase. The values reflect the mean for five gels generated from five biological replicates. The data were averaged, graphed, and statistically analyzed by two-way analysis of variance followed by the Bonferroni posttest. Asterisks or number symbols indicate a statistically significant difference relative to the results for the wild type or the *pbgA* mutant, respectively. *, *P* < 0.05; **, *P* < 0.01; ***, *P* < 0.001; ****, *P* < 0.0001; no asterisk indicates not significant.

To examine PbgA’s role in LPS homeostasis, we extracted LPS from whole bacteria or their membrane fractions. Consistent with published evidence, *S*. Typhimurium qualitatively increased its long-chain LPS modalities in stationary phase compared to log phase (Fig. 4A) (24). The wild-type and the *pbgA*Δ*191–586* mutant salmonellae produced comparable levels of the short, the long, and the very long LPS modalities in both growth phases, indicating that PbgA is not necessary for LPS synthesis (Fig. 4A). The periplasmic domain of PbgA was also not necessary for *S*. Typhimurium to transport LPS molecules to the OM, since the *pbgA* mutants and wild-type salmonellae produced similar levels of each LPS modality in their isolated OM fractions (Fig. 4B). Dramatic differences existed between *pbgA* mutants and the wild type in the level of truncated LPS precursors lacking O antigens (Fig. 4A to F). In particular, *pbgA* mutant *S*. Typhimurium accumulated what were likely lipid A-core molecules, since they migrated slightly slower than the smaller truncated lipid A-inner core molecules produced by the *S*. Typhimurium *galE* mutant (Fig. 4A, C, and D) (67). The lipid A-core levels for the *pbgA* mutants were 2- to 4-fold greater than those for the wild type and the complemented mutants in stationary phase (Fig. 4E). Modest variations were also measured for the *pbgA* mutants in the log phase of growth compared to the wild type. For instance, PbgA overexpression routinely caused the level of short-chain LPS molecules to increase in the log phase (Fig. 4A and F). Also, the *pbgA* mutants had modestly elevated levels of lipid A-core in log phase, supporting a lesser role for the periplasmic domain under replete conditions (Fig. 4E and F).

**FIG 5:**
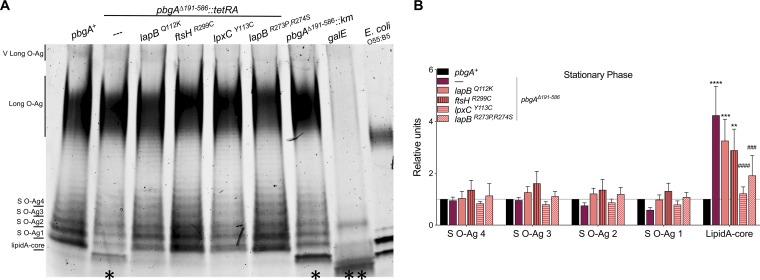
The accumulation of lipid A-core molecules by the *pbgA* mutant of *S*. Typhimurium is generally suppressed by the substitutions in LapB, FtsH, and LpxC. (A) Hot phenol extraction of LPS was performed on normalized stationary-phase cells of the *pbgA^+^* strain, two independently generated *pbgA* mutants with distinct resistance cassettes (the *pbgA*Δ*191–586*::*tetRA* and *pbgA*Δ*191–586*::*km* strains), the strains with the suppressor genotypes, and the Δ*galE* mutant. In parallel, commercial E. coli LPS (catalog number P30635; Invitrogen) was assessed. ProQ300 Emerald-stained gels were imaged using Image Lab software. The image represents that from one of five independent experiments. (B) Band intensities were measured from ProQ300 Emerald-stained gels using Image Lab software. Mutant bands were normalized to the corresponding wild-type bands. The values reflect the mean for three gels generated from three biological replicates. A two-way analysis of variance followed by the Bonferroni posttest was applied. Asterisks or number symbols indicate a statistically significant difference relative to the results for the wild type or the *pbgA* mutant, respectively. *, *P* < 0.05; **, *P* < 0.01; ***, *P* < 0.001; ****, *P* < 0.0001; no asterisk indicates not significant.

Given that the suppressive SNPs mapped to essential regulators of LPS biosynthesis, it was reasonable to predict that the suppressor isolates might have decreased lipid A-core levels relative to bacteria of the *pbgA* mutant parental genotype. Indeed, the lipid A-core levels for each suppressor were significantly less than those for the *pbgA* mutants (Fig. 5A and B). The LpxC substitution and the dual substitutions in LapB were sufficient to significantly reduce the lipid A-core levels to those for the wild type. In contrast, the single substitutions in LapB and FtsH were not fully sufficient to reduce the levels (Fig. 5A and B). Thus, *S*. Typhimurium relies upon the periplasmic domain of PbgA to regulate LPS assembly during stress, and the defect can be variably overcome by amino acid substitutions in conserved essential regulators of LPS synthesis.

### The *S*. Typhimurium PhoPQ regulators activate increases in short-chain LPS molecules by a mechanism that involves PbgA.

The globular region of PbgA is necessary for *S*. Typhimurium to establish the barrier that is erected in response to PhoPQ activation (50). Activation of the *S*. Typhimurium PhoPQ regulators increases the level of short-chain LPS modalities produced (23, 68–70). Therefore, we asked if the globular region is involved in PhoPQ-regulated LPS assembly for *S*. Typhimurium.

**FIG 6:**
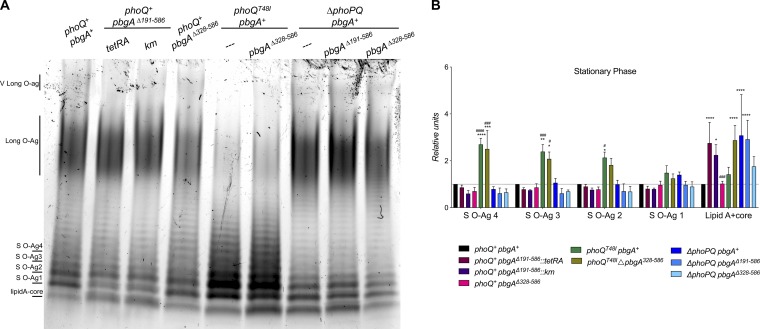
*S*. Typhimurium PhoPQ positively regulates the levels of short-chain LPS molecules at the expense of long-chain LPS molecules in a manner that involves PbgA. (A) LPS was extracted by the hot phenol method from fresh whole cells at stationary phase, separated under denaturing conditions, and visualized with ProQ300 Emerald stain. The picture represents that for one out of three biological replicates assessed for each genotype. The *S*. Typhimurium strain expressing the *phoQT48I* alleles is constitutively activated for PhoPQ signaling, while the two regulators, which are adjacent on the chromosome, were deleted from the *S*. Typhimurium Δ*phoPQ* mutant. (B) Band intensities on ProQ300 Emerald-stained gels were measured using Image Lab software (Bio-Rad). The values reflect the mean for three gels generated from three biological replicates. Mutant bands were normalized to the wild-type bands. Data were averaged and analyzed by two-way analysis of variance followed by the Bonferroni posttest. Asterisks or number symbols indicate a statistically significant difference relative to the results for the wild type or the *pbgA* mutant, respectively. *, *P* < 0.05; **, *P* < 0.01; ***, *P* < 0.001; ****, *P* < 0.0001; no asterisk indicates not significant.

As observed previously, constitutive PhoPQ activation resulted in *S*. Typhimurium increasing its short-chain LPS modalities, seemingly at the expense of its long-chain LPS modalities (Fig. 6) (23, 70). This regulation was PhoPQ dependent, since Δ*phoPQ* mutants displayed a typical distribution of LPS subtypes which resembled that of the wild type (Fig. 6). Consistent with PhoPQ and PbgA cooperatively influencing LPS assembly, the *pbgA*Δ*191–586* and Δ*phoPQ* mutant *S*. Typhimurium strains had statistically significantly equivalent lipid A-core levels that were each 3 to 4 times greater than those for the wild type (Fig. 6). Further, the PhoPQ-activated salmonellae in which the PbgA globular region was deleted (the *phoQT48I pbgA*Δ*328-586* mutant) had 2- to 3-fold greater lipid A-core levels than the PhoPQ-activated bacteria expressing wild-type PbgA (the *phoQT48I pbgA^+^* mutant) (Fig. 6). The PhoPQ-activated *pbgA*Δ*191–586* salmonellae were severely attenuated, but they, too, had increased lipid A-core levels relative to the control bacteria (Fig. S5). The LPS analysis showed that *S*. Typhimurium relies on the periplasmic domain of PbgA to properly control the assembly of the LPS glycolipid during stress.

### *S*. Typhimurium uses PbgA to control the phospholipid content of the cell envelope.

Bacteria coregulate phospholipids and LPS molecules to maintain, remodel, and repair their barrier. Phospholipid and LPS biosynthesis pathways draw from similar fatty acid resources, such as the fatty acids with a carbon length of 14. *Enterobacteriaceae* rely upon the LapB-FtsH-LpxC control axis to influence both pathways (16, 61, 71–74). The phospholipid levels for the *pbgA*Δ*191–586* mutants had not been previously determined (50). Therefore, we cultured bacteria to the log or the stationary phase and isolated their total membrane and OM fractions (Tables S3 and S4). Bacterial phospholipids were extracted and analyzed by normal-phase liquid-chromatography (LC)-tandem mass spectrometry (MS/MS) (48). The levels (in nanograms per microliter) were quantified for at least four individual species from each major head group family.

**FIG 7:**
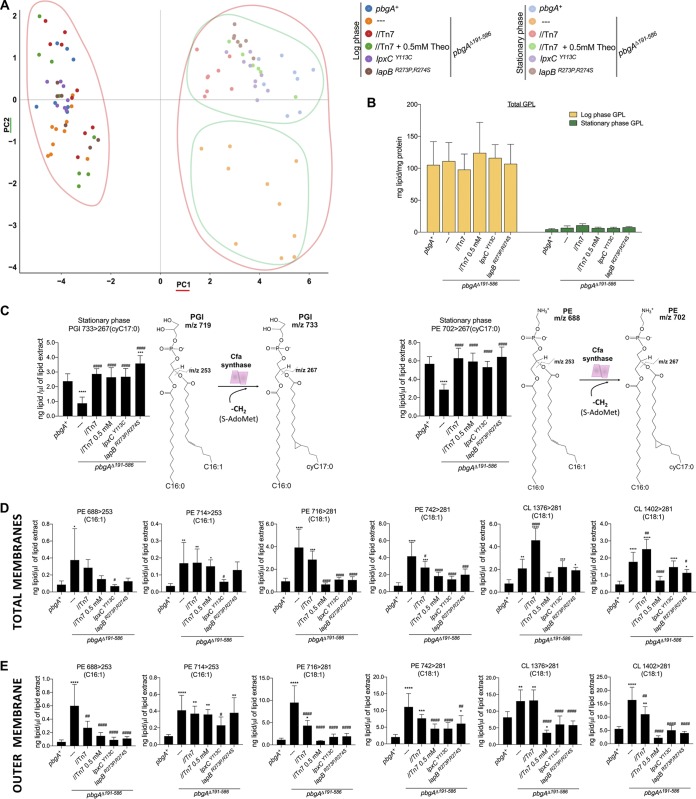
*S*. Typhimurium decreases phospholipid levels in stationary phase, and PbgA is necessary for the formation of specific cyclopropanated phospholipids. (A) Quantitative liquid chromatography (LC)-tandem mass spectrometry (MS/MS) was performed on total membrane phospholipid extracts (see Table S3 in the supplemental material). At least eight independent total membrane extracts were assessed for each genotype in each growth phase. The values (in number of nanograms of lipid per microliter of lipid extract) were analyzed by principal-component analysis (PCA). The PCA score plot axes show PC1 as the maximum variation and minimum error among data points and PC2 as the next-largest variation. Each data point in the plot represents the phospholipid profile for each sample and is color coded by genotype and growth phase. (B) The concentrations of four representative acyl-PGls, PGls, CLs, and PEs were summed to determine the number of milligrams of total lipid per milligram of total protein ± SD for each membrane sample (Table S3). At least eight independent biological replicates were analyzed per genotype and condition. (C) Analysis of the PCA eigenvectors showed two major GPL contributors of variance (PGl *m/z* 733 and PE *m/z* 702) between the samples. The levels for these specific phospholipids (in number of nanograms of lipid per microliter of lipid extract) were analyzed for the stationary-phase membranes. A one-way analysis of variance followed by the Bonferroni posttest was used to test significance. Asterisks or number symbols indicate a statistically significant difference relative to the results for the wild type or the *pbgA* mutant, respectively. Predicted structures for PGl *m/z* 719 (C_16:0_, C_16:1_), PGl *m/z* 733 (C_16:0_, cyC_17:0_), PE *m/z* 688 (C_16:0_, C_16:1_), and PE *m/z* 702 (C_16:0_, cyC_17:0_) including the characteristic daughter ions, *m/z* 253 and *m/z* 267, before and after the cyclopropane fatty acid synthase (Cfa) reaction, respectively, are depicted. (D and E) Phospholipid levels after LC-MS/MS analysis of total membranes (D) and outer membranes (E) (PE *m/z* 688, PE *m/z* 714, PE *m/z* 716, PE *m/z* 742, CL *m/z* 1,376, and CL *m/z* 1,402) are presented as the number of nanograms of lipid per microliter of extract for both the total (total) and outer membrane (bottom) samples. A one-way analysis of variance followed by the Bonferroni posttest was used to test significance. Asterisks or number symbols indicate a significant difference relative to the results for the wild type (*pbgA^+^*) or the *pbgA* mutant, respectively. OVN, overnight.

Unsupervised principal-component analysis (PCA) was applied to determine significant differences between the samples (Fig. 7A). The log- and stationary-phase samples separated across principal component 1 (PC1), which quantitatively reflected a wholesale decrease in phospholipids in stationary phase (Fig. 7A and B). The periplasmic domain of PbgA does not contribute to the ability of *S*. Typhimurium to generally decrease phospholipids in stationary phase. However, the stationary-phase *pbgA* mutant membranes clustered separately from all other samples along PC2, suggesting that significant differences existed (Fig. 7A). Indeed, we determined that the major phospholipid contributors to the PC2 variation were PGl *m/z* 733 and PE *m/z* 702 (Fig. 7C; Fig. S6; Table S3). Intriguingly, each of these phospholipids harbors a 17-carbon cyclopropanated fatty acid (cyC_17:0_; *m/z* 267) at the *sn-2* position (Fig. 7C). Likewise, each cyclopropanated phospholipid was highly abundant in the membranes of stationary-phase *S*. Typhimurium (Fig. S7). Quantitatively, the levels of PGl *m/z* 733 and PE *m/z* 702 (in nanograms per microliter) were significantly reduced in the membranes for the *pbgA*Δ*191–586* mutants compared to the wild type (Fig. 7C; Table S3). Complementation and suppression fully restored the levels of these molecules (Fig. 7A and C). The data support the suggestion that PbgA promotes cyclopropane ring formation on phospholipids during stress and that the mechanism can be bypassed by amino acid substitutions in LpxC and LapB.

The *pbgA* mutants showed additional significant differences in phospholipid levels relative to the wild type in stationary phase; however, these variations were not the major contributors to the PC2 variation (Table S3). The levels of several PEs and multiple CLs were increased in the total membrane and OM fractions of *pbgA* mutants in stationary phase compared to those in the wild type (Fig. 7D and E; Table S3 and S4). The general increase in PEs and CLs suggested that removing the periplasmic domain caused phospholipids to become dysregulated.

In the phenotypic assays described above, complementation and suppression were nearly fully sufficient to revive *pbgA* mutant attenuation (Fig. 2A to C, 3A, 4A and E, and 7A and C). However, basal PbgA expression did not significantly change the PE or CL levels for the mutants (Fig. 7D and E). In contrast, PbgA overexpression significantly reduced the PE and CL levels for the mutants (Fig. 7D and E). Further, the suppressor isolate with the substitution in LpxC and the isolate with the two substitutions in LapB also had significantly reduced PE and CL levels compared to the mutant (Fig. 7D and E). Therefore, *S*. Typhimurium uses PbgA to balance lipid homeostasis during stress in a manner that preserves OM barrier integrity.

### The *pbgA* mutant defects in Rif resistance, survival in macrophages, and survival in mice can be variably overcome by the substitutions in LapB, FtsH, and LpxC.

**FIG 8:**
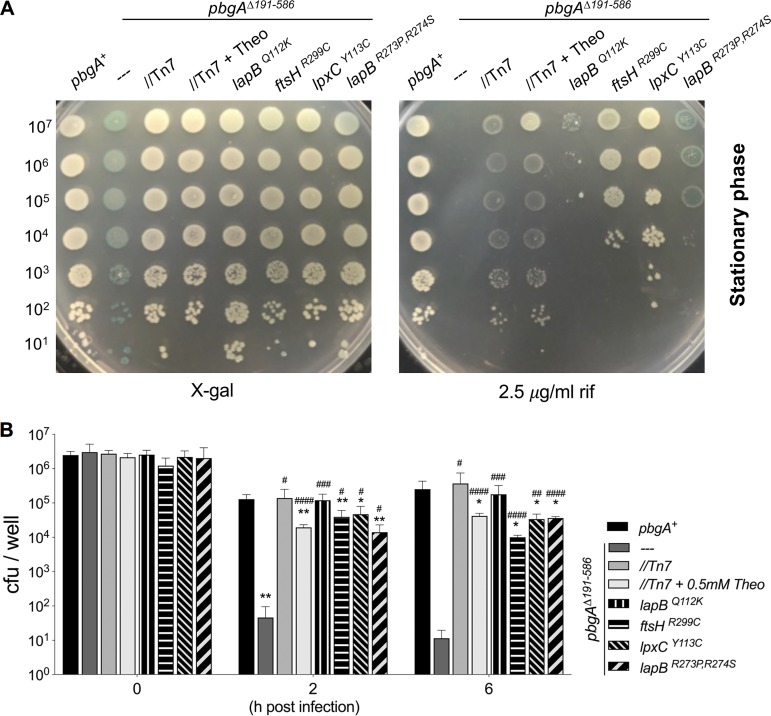
The suppressive SNPs variably restore *pbgA* mutant rifampin resistance and survival in macrophages. (A) Rifampin (Rif) susceptibility was measured by normalizing log- and stationary-phase broth-grown bacteria to an optical density at 600 nm of 1.0, serially diluting, plating 2-μl spots onto LB agar–X-Gal (5-bromo-4-chloro-3-indolyl-β-d-galactopyranoside) with and without Rif at 2.5 μg/ml, and incubating at 37°C overnight. The picture shown is representative of the pictures from three independent experiments. (B) Primary bone marrow-derived macrophages (BMDMϕs) were isolated from C57BL/6J mice and infected with the stationary-phase wild-type salmonellae, the *pbgA* mutant, the strain with the complemented genotype, or the suppressor variants each at a multiplicity of infection (MOI) of 10:1. Each strain was assayed in triplicate wells in each experiment. Lysing and plating at 2 and 6 h postinfection were used to quantify intracellular survival. Data are shown as the mean number of CFU per well ± SD and represent those from at least five independent experiments. Asterisks or number symbols indicate a statistically significant difference relative to the results for the wild type or the *pbgA* mutant, respectively.

Subtle phenotypic variations existed between suppressor isolates (Fig. 2B and C, 3A, and 5A and B). For instance, the suppressors carrying the FtsH substitution only partially restored the RcsF activation phenotype in broth culture, while the suppressors carrying the substitutions in LapB and LpxC fully resolved the defect (Fig. 2B and C). Rifampin (Rif) is a hydrophobic antibiotic that crosses the OM by diffusing through the fatty acyl components of the bilayer. Increased amounts of phospholipids and lipid A-core structures within the OM cause *S*. Typhimurium Rif sensitivity to increase dramatically (9, 58). The *pbgA* mutants were severely attenuated in the presence of Rif, and complementation generally restored their resistance (Fig. 8A). The single substitution in LapB did not improve Rif resistance, but the double substitution did, albeit modestly (Fig. 8A). The FtsH mutation improved Rif resistance to a greater degree than the LapB mutations, but the LpxC substitution conferred the greatest level of Rif resistance to the *pbgA* mutant *S*. Typhimurium. Nevertheless, no mutation was sufficient to fully restore the defect (Fig. 8A). Therefore, each suppressor had restored OM barrier integrity to the *pbgA* mutant genotype by a distinct convergent mechanism.

To quantify the ability of the suppressor mutations to restore the intracellular survival defect of the *pbgA* mutant of *S*. Typhimurium, we infected primary C57BL/6J mouse macrophages (50). By 2 and 6 h, several log fewer intracellular *pbgA* mutant salmonellae than wild-type salmonellae were enumerated (Fig. 8B; Fig. S8). Basal expression of PbgA fully restored the mutant survival defect, but overexpression did not. Unlike Rif resistance, the single substitution in LapB fully restored the intracellular survival phenotype of the *pbgA* mutants (Fig. 8B; Fig. S8). The other suppressors had numbers of CFU that were significantly greater than those of the *pbgA* mutants by multiple orders of magnitude, but they were still significantly less than those of the wild type.

*S*. Typhimurium survives within the vacuoles of macrophages to colonize the spleens and livers of mice (2). Therefore, we intraperitoneally injected male and female C57BL/6J mice with 10^5^ CFU of the suppressor mutant salmonellae and compared the surviving numbers of CFU per g of tissue organ to those for the wild type and the *pbgA* mutants at 2 days postinfection (Fig. 3A; Fig. S4). Animals infected with the suppressors were colonized with significantly higher titers than animals infected with the *pbgA* mutants (Fig. 3A; Fig. S4). However, the levels for the suppressor mutants were significantly less than those for the wild type. Therefore, although the substitutions in LapB, FtsH, and LpxC commonly restored the *pbgA* mutant defects, variations existed, and none of the suppressor isolates had fully resolved the attenuation.

### *S*. Typhimurium requires the periplasmic domain of PbgA to survive, proliferate, and cause lethality during bacteremia in mice.

The LpxC substitution routinely restored the *pbgA* mutant defects to levels that were either statistically significantly equivalent to or slightly less than those for the wild type, including the ability of the mutant to colonize mice (Fig. 2B and C, 3A, 5A and B, 7, and 8; Fig. S4). Wild-type C57BL/6J mice die within roughly 1 week after being intraperitoneally infected with low-dose inocula of wild-type *S*. Typhimurium. Therefore, we decided to compare the toxicity of the wild type, the *pbgA* mutant, and the *pbgA-lpxC* suppressor toward these animals.

**FIG 9:**
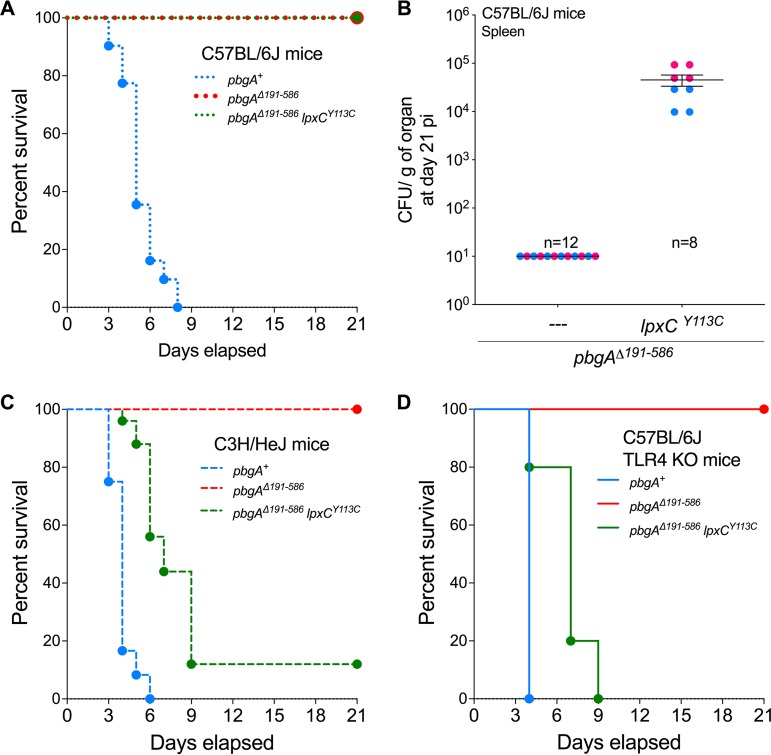
*S*. Typhimurium requires the periplasmic domain of PbgA to proliferate and cause lethality in mice, and the *pbgA-lpxC* suppressor persists in animals in a manner that involves Toll-like receptor 4. (A) The survival of C57BL/6J mice was monitored for 21 days after i.p. infection with roughly 10^3^ CFU of the *pbgA^+^* strain (*n* = 33), *pbgA* mutant (*n* = 12), or the *pbgA lpxC* suppressor variant (*n* = 31) (some strains were tested in a higher number of mice to ensure accuracy). Data represent those from four independent experiments and are shown as percent survival. Median survival was 5 days, 21 days, and 21 days for mice inoculated with the *pbgA^+^* strain, the *pbgA* mutant, and the *pbgA lpxC* strain, respectively. Comparison of the curves was done using the log-rank (Mantel-Cox) test (*P* < 0.0001). (B) Enumeration of the number of CFU per gram of spleen ± SEM for C57BL/6J mice infected with the *pbgA* mutant (*n* = 12 mice) or the *pbgA lpxC* suppressor variant (*n* = 8), and any surviving mice were sacrificed at 21 days postinfection (pi). (C) The survival of C3H/HeJ mice was similarly assessed. The data represent those from three experiments. The median survival times were 4 days, 21 days, and 7 days for mice inoculated with the *pbgA^+^* strain (*n* = 12 mice), the *pbgA* mutant (*n* = 14), and the *pbgA lpxC* strain (*n* = 25), respectively (some strains were tested in a higher number of mice to ensure accuracy). A log-rank (Mantel-Cox) test was used. The *P* value was <0.0001 when comparing the *pbgA*^+^ strain, the *pbgA* mutant, and the strain with the *pbgA lpxC* genotype. The *P* value was <0.0001 when comparing the *pbgA*^+^ and the *pbgA lpxC* bacteria. (D) The survival of C57BL/6J Toll-like receptor 4 (TLR4)-knockout mice was similarly assessed. The data represent those from three experiments. The median survival times were 4 days, 21 days, and 7 days in mice inoculated with the *pbgA^+^* strain (*n* = 5 mice), the *pbgA* mutant (*n* = 5), and the *pbgA lpxC* strain (*n* = 5), respectively (some strains were tested in a higher number of mice to ensure accuracy). A log-rank (Mantel-Cox) test was used. The *P* value was <0.0001 when comparing the results for mice inoculated with the *pbgA*^+^ strain, the *pbgA* mutant, and the strain with the *pbgA lpxC* genotype. The *P* value was <0.0001 when comparing the results for mice inoculated with the *pbgA*^+^ and the *pbgA lpxC* bacteria.

We intraperitoneally inoculated C57BL/6J animals with roughly 10^3^ salmonellae and monitored them until they were moribund or at 21 days postinfection. Mice inoculated with the wild-type salmonellae succumbed by 8 days and yielded roughly 10^7^ to 10^10^ CFU/g of tissue in the livers and spleens (Fig. 9A; Fig. S9A). Mice infected with the *pbgA* mutants were asymptomatic, did not perish, and yielded zero surviving salmonellae at 21 days (Fig. 9A and B; Fig. S9A). Interestingly, the mice infected with the *pbgA-lpxC* suppressors were also asymptomatic and did not succumb. However, these animals remained persistently colonized for months with between 10^3^ and 10^4^ CFU/g of the *pbgA-lpxC* mutant salmonellae (Fig. 9A and B; Fig. S9B and S10A). Given the role of PbgA and LpxC in maintaining LPS homeostasis, we reasoned that mouse TLR4 might be necessary to control the persistent infections.

### Mice require TLR4 to restrict the proliferation and toxicity of the *S*. Typhimurium *pbgA-lpxC* mutant.

Two mouse strains with mutations in *tlr4* were infected with *S*. Typhimurium to test whether TLR4 influences the persistence phenotype achieved by the *pbgA-lpxC* suppressor salmonellae (Fig. 5A and B and 8A). The genomes of C3H/HeJ animals encode a substitution mutation in TLR4 that renders the receptor insensitive to activation by the lipid A endotoxin (75). The C57BL/6J TLR4-knockout mice had a *tlr4* deletion. Each TLR4-deficient mouse strain succumbed to wild-type *S*. Typhimurium infections within 5 days but survived *pbgA* mutant infections and resolved the infection with the salmonellae by day 21 (Fig. 9C and D; Fig. S9B and S10B). The majority of the C3H/HeJ animals and all of the TLR4-knockout animals expired from *pbgA-lpxC* infections within roughly 1 week. These mice were colonized with between 10^6^ and 10^9^ CFU/g at the day of death (Fig. 9C and D; Fig. S9B and S10B). Therefore, in the context of a TLR4-deficient host, the *pbgA-lpxC* suppressor is nearly as virulent as the wild-type salmonellae (Fig. 9B; Fig. S10B). Therefore, mouse TLR4 is necessary to restrict *S*. Typhimurium proliferation and promote host survival during infection with the evolved *pbgA-lpxC* suppressor variants.

## DISCUSSION

**FIG 10:**
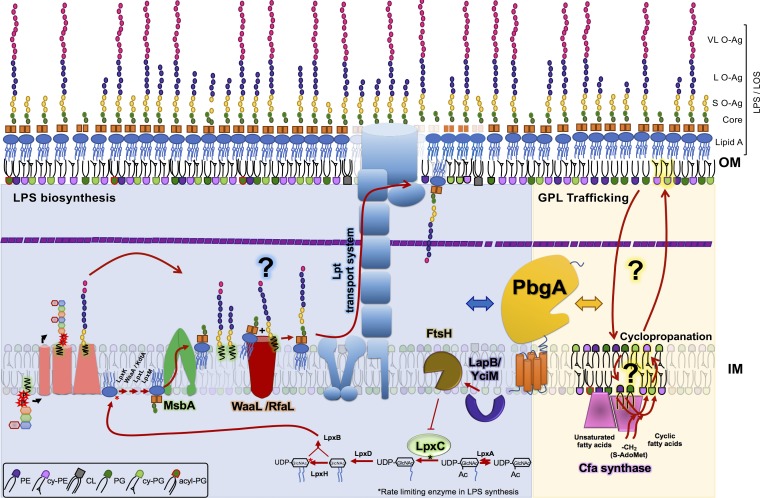
Model showing how *S*. Typhimurium requires PbgA to control phospholipid trafficking and LPS assembly during stress. The model generally places PbgA in a central regulatory role that impacts both LPS assembly and phospholipid trafficking within the dual-bilayered cell envelope of *S*. Typhimurium. Further details for the individual components have been previously overviewed, and the predictions and details regarding the model are included in the Discussion (Fig. 1 and 3C).

The OM is a formidable barrier that protects Gram-negative bacteria against antimicrobial agents and immune systems. *S*. Typhimurium uses the periplasmic domain of the IM protein PbgA to promote OM integrity, resistance to antibiotics, and pathogenesis for mice (Fig. 2, 3A, 4, 8, and 9) (50). To investigate PbgA’s involvement in promoting *S*. Typhimurium LPS integrity, we studied *pbgA* mutants in which the nonessential periplasmic domain of the PbgA protein was deleted but in which the essential transmembrane domain was retained (50, 52). Our results show that *S*. Typhimurium relies on PbgA to regulate LPS assembly and that this is necessary for salmonellae to survive and proliferate during bacteremia in mice. Several mechanisms are plausible. For instance, PbgA could bind Und-PP–O antigens, lipid A-core molecules, or the WaaL ligase (Fig. 10). Alternatively, salmonellae might indirectly require PbgA to regulate LPS assembly by facilitating phospholipid movement between or across the bilayers (Fig. 10) (50). Moreover, it is conceivable that salmonellae use the periplasmic domain of PbgA to detect or transmit lipid signals during stress.

E. coli increases its LapB levels during stress, and LapB responds to an undefined fatty acid signal that triggers its binding to FtsH. LapB-FtsH interactions prompt the degradation of LpxC (71, 74, 76). In this model, LapB negatively regulates LPS biosynthesis in response to stress (Fig. 3C). We envision that *S*. Typhimurium uses LapB to bind PbgA or a PbgA-associated lipid molecule (Fig. 10). This might allow salmonellae to detect changes in lipid homeostasis and metabolism. We posit that *S*. Typhimurium uses the periplasmic domain of PbgA to facilitate lipid trafficking during stress (Fig. 10). We predict that the transmembrane segments execute an undefined essential activity involving phospholipids. Together with the LapB-FtsH-LpxC control axis, *S*. Typhimurium relies upon PbgA to balance the lipid composition of the cell. Ultimately, the two domains of PbgA must work in concert for *S*. Typhimurium to govern lipid homeostasis during bacteremia.

### What is the mechanism by which PbgA impacts LPS assembly?

Our LPS measurements are only semiquantitative; however, the *pbgA*Δ*191–586* mutants did not obviously lack or overproduce any particular LPS modality, yet they accumulated lipid A-core molecules within their OMs (Fig. 4A to F). Thus, the periplasmic domain is not necessary for *S*. Typhimurium to synthesize, assemble, or polymerize Und-PP–O antigens and is also not required for salmonellae to synthesize and assemble lipid A-core molecules. Further, the PbgA periplasmic domain is dispensable for *S*. Typhimurium to transport LPS or lipid A-core molecules to the OM (Fig. 4B). Therefore, *S*. Typhimurium must rely on PbgA to regulate LPS assembly.

*S*. Typhimurium regulates its LPS modalities during stress, and this requires the periplasmic domain of PbgA (Fig. 4A to F and 6). The PhoPQ regulators are modestly activated in stationary-phase cultures, yet the LPS profile of stationary-phase *S*. Typhimurium varies dramatically from that of bacteria in which PhoPQ is activated (Fig. 6). Therefore, additional stationary-phase regulators likely rely upon PbgA to influence LPS assembly and phospholipid homeostasis. In the humanized mouse model of typhoid fever, the PhoPQ regulators are dispensable for *S*. Typhi virulence, while the periplasmic domain of PbgA is critical (77). Therefore, we speculate that additional S. enterica regulators require PbgA to control lipid homeostasis during systemic diseases caused by salmonella serovars.

If PbgA directly influences the ligation reaction catalyzed by WaaL, then *pbgA* mutants should also accumulate Und-PP–O antigens during stress. If *pbgA* mutants accumulate Und-PP–O antigens, it is plausible that the UDP available to other pathways becomes diminished. For example, peptidoglycan biosynthesis requires Und-PP to move precursor intermediates across the IM (78, 79). Under certain conditions, *pbgA*Δ*191–586* mutants are morphologically altered and show defects in septation, suggesting a possible connection between PbgA and peptidoglycan (see Fig. S5B in the supplemental material) (50). PbgA binds phospholipids with a greater affinity for doubly charged CL anions than for singly charged PGl anions and PE zwitterions (50). Like CL anions, Und-PP–O antigens and lipid A-core molecules possess dual phosphates in their structures. Therefore, it is reasonable to predict that the basic region of PbgA might bind to these molecules (Fig. 10). Regardless of the ligand, the lipid-binding residues of PbgA are necessary for the *S*. Typhimurium OM barrier function (50).

### What is the mechanism by which PbgA influences phospholipid levels within the envelope?

*S*. Typhimurium requires the globular region of the PbgA periplasmic domain to increase the OM CL content during constitutive activation of the PhoPQ regulators (50). Therefore, it was reasonable to predict that PbgA might promote phospholipid trafficking between the bilayers. The results here support the possibility that PbgA promotes the formation of particular cyclopropanated phospholipids, PGl *m/z* 733 and PE *m/z* 702, during stationary phase (Fig. 7). Since phospholipid cyclopropanation necessitates trafficking between and across the bilayers, the role of PbgA might be associated with returning the monounsaturated precursors to the cytosolic leaflet of IM (Fig. 10) (35, 36).

During stationary-phase stress, *S*. Typhimurium *pbgA*Δ*191–586* mutants accumulate CL and PE throughout their membranes (Fig. 7D and E). The original E. coli *pbgA* (*yejM*) LH530 truncation mutant produced a truncated PbgA polypeptide that terminated at residue 190 (52, 80, 81). During heat shock, the mutant had increased levels of fatty acid incorporation into phospholipids relative to LPS molecules in comparison to those for the wild type (80, 81). Therefore, our data are consistent with past evidence and suggest that the periplasmic domain of PbgA is necessary for *S*. Typhimurium to balance LPS and phospholipid homeostasis within the OM (Fig. 10).

Given the essential nature of the transmembrane segments and the demand for PbgA during stress, the deletion likely has pleiotropic consequences. Accordingly, some biochemical phenotypes of *pbgA* mutants might be indirect or the result of the expression of a truncated transmembrane domain that lacks its periplasmic partner. Loss of the PbgA periplasmic domain causes *S*. Typhimurium to increase its CL and PE levels throughout the envelope (Fig. 7D and E). Expressing PbgA from the neutral locus in the absence of theophylline was fully sufficient to increase the levels of the cyclopropanated phospholipids and decrease the lipid A-core levels for the *pbgA*Δ*191–586* mutants; however, basal PbgA expression did not change the PE and CL levels (Fig. 4A and C and 7C to E). *pbgA* mutants that overexpressed PbgA, encoded the substitution in LpxC, or encoded dual substitutions in LapB each had statistically significantly decreased PE and CL levels compared to the *pbgA* mutants, in which the levels were similar to those in the wild type (Fig. 7C to E). Given that full rescue necessitated overexpression or extragenic suppression, it is possible that these lipid changes are a pleiotropic consequence of the deletion of this region. Nevertheless, the increase in PE and CL phospholipids within the membranes of the complemented mutant expressing basal PbgA levels did not have profound phenotypic effects, since the mutants were almost fully rescued for all phenotypes (Fig. 2A-B, 4A and C, 7C, and 8). The data support the suggestion that the periplasmic domain of PbgA is not required for *S*. Typhimurium to export CL to the OM, but the results do not exclude the possibility of a role for PbgA in promoting OM-CL homeostasis. Future experiments will be necessary to determine the exact mechanisms of PbgA-mediated phospholipid alterations and their impact on host-pathogen interactions.

### How do the lipid alterations influence the outcome of pathogenesis?

The gross OM integrity defect for the *pbgA* mutant of *S*. Typhimurium can likely be explained, in part, by the overaccumulation of lipid A-core molecules within the OM outer leaflet and the overabundance of phospholipids within the inner leaflet (Fig. 4 to 6). *S*. Typhimurium mutants defective for O-antigen synthesis are avirulent in mouse models of disease and are hypersensitive to hydrophobic antibiotics and serum complement (26, 82). However, unlike the classically studied rough mutants, *pbgA* mutants still produce equivalent levels of all major LPS subtypes (Fig. 4A and 5A). Patches of lipid A-core within the OM outer leaflet for *pbgA* mutants and elevated phospholipid levels within the inner leaflet would attenuate the barrier against hydrophobic antibiotics, antimicrobial peptides, and serum complement components (58, 83).

The involvement of TLR4 in the persistence phenomenon of the *pbgA-lpxC* salmonellae is biologically interesting. *S*. Typhimurium mutants defective in aromatic amino acid biosynthesis persist in C57BL/6J mice; however, the exact mechanism is not known (84). C57BL/6J mice withstand infections with the *pbgA-lpxC* bacteria and survive asymptomatically for months (Fig. S10A). Our work shows that without TLR4, mice are unable to restrict the proliferation of the *pbgA-lpxC* salmonellae and generally succumb to these infections (Table 1; Fig. 9; Fig. S9 and S10). It will be interesting to determine whether a particular PbgA-associated phospholipid or LPS precursor molecule impacts the TLR4 response to *S*. Typhimurium.

This work highlights the critical role of bacterial lipids for antimicrobial resistance and disease pathogenesis and unveils key features of *S*. Typhimurium membrane lipid regulation that are critical during stress. Our models can be probed in other Gram-negative pathogens that possess PbgA/YejM to define conserved mechanisms that might be targets for future antimicrobials.

## MATERIALS AND METHODS

### Ethics statement.

All animal procedures were carried out with approval from the University of Oklahoma Health Sciences Center Institutional Animal Care and Use Committee under protocol number 19-015-ACI. The procedures used in this study strictly adhered to the guidelines found in the National Research Council’s Guide for the Care and Use of Laboratory Animals (86).

### Bacterial strains and culturing conditions.

The bacterial strains used in this study were derivatives of the Salmonella enterica serovar Typhimurium genotype 14028s strain, which contains a chromosomally integrated *wza-lacZ* gene promoter fusion (85) (see Table S1 and the Materials and Methods section in the supplemental material for additional details).

### Genetics.

The details for generating the *pbgA*Δ*191–586*::*tetRA* deletion-insertion genotype have been described previously (50) (see the Materials and Methods section in the supplemental material for additional details).

### Growth curves.

Three growth curves were generated using small (170-μl), medium (5-ml), and large (1-liter) culture volumes to assess the growth phases. In all cases, the inoculum consisted of a single resuspended bacterial colony. The growth curve for the 170-μl culture volume was generated in a Bioscreen C growth curve analyzer (see the Materials and Methods section in the supplemental material for additional details).

Also see the Materials and Methods section in the supplemental material for additional details on the β-galactosidase (β-Gal) assay, rifampin sensitivity assay, and whole-genome sequencing.

### Murine macrophage infections.

Primary bone marrow-derived murine macrophages (BMDMϕs) were prepared by harvesting the marrow from the femurs of 6- to 8-week-old C57BL/6J L/6 mice from The Jackson Laboratory. See the Materials and Methods section in the supplemental material for additional details.

Also see the Materials and Methods section in the supplemental material for additional details on membrane fractionation, protein quantification and glycerophospholipid (GPL) extraction, clearing of anti-PbgA rabbit sera, Western blotting, and normal-phase liquid chromatography (LC) electrospray ionization-tandem mass spectrometry (MS/MS).

### Mouse infections.

Male and female C57BL/6J L/6 and C3H/HeJ mice were purchased from The Jackson Laboratory and bred in-house under pathogen-free conditions. To measure the ability of *S*. Typhimurium to survive systemically and colonize the spleens and livers of mice, 6- to 8-week-old mice were intraperitoneally (i.p.) infected with roughly 5 × 10^5^ CFU diluted in PBS. At 48 h, the mice were euthanized and the livers and spleens were dissected, weighed, and homogenized in phosphate-buffered saline–0.1% Triton X-100 (see the Materials and Methods section in the supplemental material for additional details).

Also see the Materials and Methods section in the supplemental material for additional details on lipopolysaccharide extraction, electrophoresis, and the detection and semiquantification of short-O-antigen LPS molecules.

### Statistical analysis.

All statistical analyses were performed and graphs were prepared using Prism (version 8) software (GraphPad Software, La Jolla, CA, USA). Principal-component analysis (PCA) was executed using the BioVinci platform with data that were log transformed (BioTuring, San Diego, CA, USA).

## Supplementary Material

Supplemental file 1

Supplemental file 2

Supplemental file 3
