# Supplementary material for: Salmonella enterica Serovar Typhimurium Uses PbgA/YejM To Regulate Lipopolysaccharide Assembly during Bacteremia
Source: Infect Immun. 2019 Dec 17;88(1):e00758-19. doi: 10.1128/IAI.00758-19 (PMC6921655; doi:10.1128/IAI.00758-19)
Supplement: Supplemental file 1 [file IAI.00758-19-s0001.pdf]

SUPPLEMENTARY INFORMATION TEXT

Supplemental materials and methods:

Bacterial strains and culturing conditions (*continued from the Main Text*). Each strain was streaked onto Luria-Bertani (Difco™) agar plates containing the LacZ-indicator substrate, 5-Bromo-4-Chloro-3-Indolyl β-D-Galactopyranoside (Xgal), at a concentration of 20μg/ml. The bacteria were incubated aerobically at 37°C overnight. Colonies were isolated from -80ºC glycerol stocks, weekly. A single colony was typically inoculated into LB-broth medium and shaken, or rotated at 250 revolutions per minute, aerobically at 37°C until the mid-exponential growth phase (*log phase*-hereafter), which is typically defined as an optical density at 600nm [OD_{600}] of 0.6 to 0.8 for a 5ml culture, or the mid-stationary growth phase (*Stat. phase*, hereafter), which we determined was achieved at 16h post single-colony inoculation (see details for generating growth curves). Culturing volumes varied depending on the phenotypic assay: for beta-galactosidase assays, macrophage and mouse infections, LPS extraction and antibiotic susceptibility plating, 5ml culture volumes were used. To collect membranes for phospholipid quantification, 1L cultures were used. Details are given in the individual assay description. The complementation genotype contains a single chromosomal copy of the wild-type *pbgA* allele at the Tn7 integration site, which is 3' of *glmS*. The T5 promoter drives transcription of *pbgA* at this neutral site (1). PbgA expression is partially repressed by a theophylline-sensitive riboswitch that is encoded between the promoter and the translational start site, and not otherwise present on the *S.* Typhimurium genome. This genotype, *pbgAΔ191-586::tetRA att::Tn7-pbgA*+ was grown in LB broth

without theophylline (Tokyo Chemical Industry) for low-level PbgA expression, and with 0.5mM theophylline for PbgA overexpression (**Fig. S3**).

Genetics (*continued for the Main Text*). To construct the chromosomal complementation genotype, *pbgAΔ191-586::tetRA att::Tn7-pbgA*+, we first used Gibson Assembly to clone the full-length PbgA coding sequence into the pBAV1k vector 3' of the T5 promoter and the riboswitch E (2, 3). The T5-riboswitch E-*pbgA* allele was amplified from the pBAV1k template, restriction enzyme digested, and ligated into pGRG37 between the left and right insertion elements of the Tn7 transposon (1). The pGRG37 vector allowed for transposition of the *pbgA* rescue allele at the neutral site 3' of *glmS*. We were initially concerned that the tetracycline resistance cassette and selection might have impacted *pbgA* mutant phenotypes and the evolution of suppressor genotypes. Therefore, we constructed an identical mutant allele using an alternative antibiotic resistance gene cassette. The *pbgAΔ191-586::kan* deletion-insertion genotype, which encoded for kanamycin resistance, was constructed using the phage lambda-red recombinase system (4). The deletion-insertion allele was then horizontally transferred into a fresh non-pKD46-bearing wild-type *wza-lacZ* salmonellae using bacteriophage P22 HT105/1 int-201. A similar procedure was used to delete *phoPQ* from the chromosome.

Growth curves (*continued for the Main Text*). The initial inoculum was prepared after resuspending a single colony in 170μl of LB broth and serial diluting 10^{-1}, 10^{-2}, and 10^{-3} 10^{-4}. Bacteria were incubated at 37°C for 24h with continuous agitation and OD_{600}

measurements were recorded at 30min time intervals. Four biological replicates were performed for each genotype and the results reflect the average. The 5ml growth curve was generated using a roller drum (**Fig. S1A**). A single colony was inoculated into 5ml of LB broth and incubated at 37°C at ~250 rpm. OD_{600} readings were taken hourly for 16h. Three biological replicates were performed per genotype and experiment. The results reflect the average for three experiments. The 1L growth curve was produced in a shaker incubator. A single colony was inoculated into 1L of LB and incubated at 37°C and ~250 rpm. OD_{600} readings were recorded hourly for 24h (**Fig. S1B**). The results reflect the average from two independent experiments.

Beta-galactosidase (β-Gal) assay (*continued for the Main Text*)**.** The β-Gal assays were performed using standard procedures. Briefly, 5ml of log- and stationary-phase bacterial cultures were pelleted and re-suspended in Z-buffer. OD_{600} readings were taken to quantify culture density. Bacteria were permeabilized with chloroform and 0.1% (wt/vol) SDS. The time to develop a yellow color was recorded following addition of ortho-Nitrophenyl-β-galactoside (ONPG) (4mg/ml in PBS) followed by incubation at 30°C. Na_2CO_3 at 1M was added to stop the reaction and the mixture was centrifuged. The supernatant was withdrawn and OD_{420} measurements were made. The levels of LacZ, or β-Gal, activity were calculated according to the formula (OD_{420})/(time x volume x OD_{600}). Three biological replicates were measured for each genotype in each experiment. The mean values were calculated from three experiments.

Rifampin sensitivity assay (*continued for the Main Text*). Plating efficiency on rifampin (Rif) was determined by first adjusting the density of 5ml log- and stationary-phase cultures to OD_{600} 1.0. Cells were serially diluted ten-fold in PBS and 2µl aliquots were spotted onto LB agar with Xgal, with or without rif at 2.5µg/ml. The plates were incubated at 37ºC OVN. The complementation genotype was grown in broth with and without 0.5mM theophylline. However, theophylline was not added to the plates. This allowed us to distinguish the effect of low-level PbgA expression and overexpression in broth culture.

Whole-genome sequencing (*continued for the Main Text*)**.** To isolate genomic DNA for sequencing, strains were grown OVN in 5ml cultures of LB broth at 37°C. Genomic DNA was isolated using a QIAamp® DNA Mini Kit. A library of random fragments was generated for each genome using standard Illumina Nextera libraries. Libraries were sequenced according to manufacturer's standards on an Illumina MiSeq system, and 300- or 600-bp paired-end reads were generated at a minimum of 35-fold genome coverage. The genomes for the wild type and the *pbgAΔ191-586::tetRA* mutant, which carried the *wza-lacZ* reporter, were sequenced and compared to the 14028s reference genome to first identify *pbgA*-independent variations that might exist in this strain background. The sequenced suppressor genomes were then compared against the mutant to identify candidate non-synonymous single nucleotide polymorphisms (snps). Additional details are provided in **Table 1**. After sequencing, the snps were individually confirmed by primer targeted sequencing of the LapB/YciM, FtsH, and LpxC coding region.

Murine macrophage infections (*continued for the Main Text*). Infected macrophages were incubated at 37°C under 5%CO_2 for 1h. The infected cells were washed and aspirated three times with phosphate-buffered saline (PBS) to remove extracellular bacteria. RPMI+FBS with 100µg/ml of gentamycin was added to kill remaining extracellular bacteria. Infected cells were incubated for an additional 1h at 37°C under 5%CO_2. At 2h post-infection (hpi), PBS+0.1% Triton was added for lysis and monolayers were gently scraped and collected with a pipette. Three wells per bacterial genotype were assessed per time point. Surviving intracellular colony-forming units (cfu) were enumerated by plating serial dilutions in PBS. After 2 hpi, the wells for the 6hpi time point were aspirated and RPMI+FBS containing 10µg/ml of gentamycin was added to kill bacteria that become extracellular during infection. At 6hpi, macrophages were lysed and surviving intracellular cfu were enumerated.

Membrane fractionation (*continued from the Main Text*). Total membranes were collected and separated as follows. Log and stationary phase cultures were normalized to OD_{600} 0.7 and harvested by centrifugation. The pellets were re-suspended in a sucrose solution to begin the lysis procedure, which allows for the efficient separation of the bilayers into two defined fractions, an IM fraction and an OM fraction (5). Briefly, the total membranes were resuspendend in 20% sucrose in 10mM Tris-HCl pH 7.8 and 0.5mM EDTA and 200µl was collected as a total membrane fraction for each genotype and stored at -20°C. The remaining 800µl of total membrane was applied to a discontinuous sucrose density gradient in order to isolate the IM and OM fractions. The

gradient consisted 2ml of 73% sucrose, 4ml of 53%, 1ml of resuspended membranes in 20% sucrose. The gradient was filled to volume with the 20% sucrose solution. Each sucrose solution was prepared in 10mM Tris-HCl pH 7.8 at 0.5mM EDTA. Density gradients were centrifuged for 16h at 35k rpm using a Beckman Optima L-90K Ultracentrifuge. Low-density IM fractions were carefully collected as a defined upper interface, brown in hue, using a pipette. High-density OMs were collected as a defined lower white interface. The membranes were washed and resuspended in 1ml of 1mM Tris, pH7.5, and stored at -20ºC

Protein quantification and phospholipid extraction (*continued for the Main Text*). Protein concentrations were measured using Pierce Coomassie Plus Bradford assay reagent (Thermo Scientific) and a standard curve generated from bovine serum albumin standard (BSA). The equivalence of 1mg of protein for each membrane was extracted by Bligh-Dyer method to obtain the phospholipid extract that was ultimately analyzed by liquid chromatography tandem mass spectrometry (LC-MS/MS) (6).

Clearing of anti-PbgA rabbit sera (*continued for the Main Text*). Antibodies to PbgA(191-586) were raised in rabbits as described previously (7)**.** 5ml of rabbit antisera was eluted over a protein-A column (Thermo) to isolate Fc/Fab fragments. Non-specific *S.* Typhimurium cross-reacting antibodies were cleared by incubating 50µL of the Fc/Fab elution fractions with 50µL of a cell lysate from *pbgA∆191-586* mutant *S.* Typhimurium in 50mM Tris-HCl pH 8.0 10mM EDTA and 5% non-fat dried milk for 4h at room temperature.

Western blotting (*continued for the Main Text*). Bradford assays were used to measure the protein concentration of the total membrane fractions. 20µg of protein was loaded onto a 10% SDS-PAGE gel, electrophoresed, and transferred onto a polyvinylidene fluoride (PVDF) membrane using a wet transfer method and the Mini Trans-Blot Cell (BioRad) at 100V for 45min. The membrane was washed and blocked overnight at 4°C in Tris-buffer saline with tween 20 (TBST) and 5% non-fat dried milk. The primary antibody was diluted 1:500 in TBST and applied to the blocked membrane and incubated overnight at 4 °C. Anti-rabbit-HRP secondary antibody was diluted 1:5000 in TBST and incubated for 1h at room temperature. Blots were imaged after detection with an Amersham ECL Western Blotting Detection Reagent (GE) using the BioRad ChemiDoc MP imager.

Phase-contrast and epifluorescence microscopy Bacteria were labeled as described previously (7). Briefly, each strain was incubated with FM4-64 (0.5 μg/ml) for 1 h at 37 °C in Luria-Bertani Broth. Next, bacteria were diluted in PBS and immobilized on 0.2% agarose pads. After gentle drying the pads, the pads were sealed within a coverslip, using a hot-glue gun. Bacteria were imaged using the appropriated UV-filters.

Normal-phase liquid chromatography electrospray ionization tandem spectrometry (LC-MS/MS) (*continued for the Main Text*). Samples were delivered to an Applied Biosystems Sciex API 4000 Triple Quad Mass Spectrometer using a Waters Acquity H-class UPLC system interfaced with an Agilent Zorbax Rx-SIL column (2.1 ×

100 mm, 1.8 μm). Analytes were ionized by electrospray in $[M-H]^{-1}$ mode with a voltage of −4.5kV. The source temperature was 450°C. Nitrogen was used as the curtain gas (setting 10), nebulizer gas (setting 20) and turbo gas (setting 20). MS/MS was performed using nitrogen as the collision gas (setting 4.0). The declustering, entrance, and collision cell exit potential was -120, -10, and -15, respectively. Retention of acyl-PGl, PGl, CL, and PE was achieved at a flow rate of 0.35 ml/min using mobile phase A [$CHCl_3$/CH_3OH/NH_4OH (800:195:5, vol/vol/vol)] and mobile phase B [$CHCl_3$/CH_3OH/H_2O/NH_4OH (600:340:50:5, vol/vol/vol/vol). A three-step gradient used started at 0% B for 1 min, continued at 0– 50% B over the next 3min, was held at 50% B for 4min, re-turned to starting conditions in 0.1min, and was allowed to equilibrate for an additional 3min, giving a total run time of ~11.1min. The following parent-to-daughter ion transitions were monitored [multiple reaction monitoring (MRM)]: 955 > 253 a-PGl, 958 > 255 a-PGl, 986 > 255 a-PGl, 1012 > 255 a-PGl, 719 > 253 PGl, 733 > 267 PGl, 747 > 281 PGl, 759 > 267 PGl, 773 > 281 PGl, 1322 > 253 CL, 1348 > 253 CL, 1376 > 281 CL, 1402 > 281 CL, 688 > 253 PE, 702 > 267 PE, 714 > 253 PE, 716 > 281 PE, 742 > 281 PE. In the case of acyl-PGl, PGl, and PE molecules, singly charged [M-H]− parent > daughter ion transitions were monitored. For CL, doubly charged [M-2H]2- parent ions were monitored because they were more abundant than singly charged ions. Collision energies ranged from -35 to -40V for the individual analytes. The data were acquired with the Sciex Analyst software version 1.6.2 (Applied Biosystems, Foster City, CA, USA).

The Bligh-Dyer phospholipid extracts from the membranes were dried under N_2 gas and resuspended in 300μl of mobile phase A. 60μl was loaded into glass inserts in

sample vials and added to the Sample Manager for the UPLC console. The flow through needle delivered 10µl injections for each extract over the column. The retention times for the major phospholipid families were confirmed and standard curves were generated using commercial standard from Avanti Polar lipids. These included 1-palmitoyl-2-oleoyl-sn-glycero-3-phosphoethanolamine, (C16:0, C18:1) PE, m/z 716.7, 1-palmitoyl-2-oleoyl-sn-glycero-3-phospho-(1'-rac-glycerol), (C16:0, C18:1) PGl, m/z 747.6, and 1',3'-bis[1-palmitoyl-2-oleoyl-sn-glycero-3-phospho]-glycerol, (C16:0, C18:1, C16:0, C18:1) $[M\text{-}H]^{-}$ CL m/z 1404 or $[M\text{-}2H]^{-2}$ m/z 702. Integrated peak areas were plotted against standard concentration to generate a linear equation. Peak area values for the targeted parent > daughter ion transitions were applied to the curve to determine the ng of the PE, PGl, and acyl-PG per µl of sample (ng/µl). At least eight independent biological replicates per strain and per growth condition (log or stationary) were examined, quantified, and averaged to give the ng/µl concentration for each individual phospholipid that we targeted within the extract.

Mouse infections (*continued from the Main Text*). Homogenates were serially diluted and plated onto LB agar with X-Gal and chloramphenicol (10µg/ml) and incubated overnight at 37°C. The antibiotic resistance cassette is linked to the *wza-lacZ* allele, so that all genotypes in this study are resistant to chloramphenicol and possess the gene reporter of Rcs-signaling activity. Colony counts were normalized to individual organ weight. For murine lethality studies, 6–8-week-old male and female mice were intraperitoneally infected with roughly 5×10^3 cfu/mouse. The weight, appearance, and behavior of each infected animal were monitored daily using our standard mouse pain,

distress and morbidity scoring system. Measurements were taken until at least 21days post infection (dpi) or until mice achieved a threshold morbidity score. The livers and spleens of deceased mice were dissected, weighed, homogenized, and plated to enumerate cfu. Randomly selected C57BL/6J mice infected with *pbgAΔ191-586* and *pbgAΔ191-586 lpxCY113C* bacteria were euthanized at day 21, to assess cfu in the livers and spleens. During experiments were the complementation genotype was tested, 0.5mM theophylline was added to the drinking water of the cage housing the infected animals.

Lipopolysaccharide (LPS) extraction, electrophoresis, and detection (*continued from the Main Text*). Log and stationary-phase cultures were normalized to OD_{600} 2.0, pelleted, and resuspended in 200μl of DEPC treated endotoxin free water. To corroborate that each sample contained approximately the same cfu level, 20μl was used for serial dilutions in PBS and 100μl aliquots were plated to enumerate bacteria concentrations in the solution. Next, 2μl of 2% SDS was added to the remaining 180μl of sample and the solution was boiled for 5min. 5ul of Proteinase K (NEB) was added to the reaction and the samples were incubated at 59ºC, overnight. Ice-cold Tris-saturated phenol was added (1:1) and the samples were vortexed and incubated at 70ºC for 15min. After cooling, diethyl ether was added (4:1) to generate a two-phase solution. The solution was centrifuged and the bottom layer was harvested and re-extracted. Samples were mixed with Laemmli buffer 4x (BIORAD) and 15μl aliquots were separated by denaturing gel electrophoresis using 4-15% polyacrylamide gradient gels (Tris-Glycine-SDS Buffer) (BIORAD) and stained with ProQ300Emerald

Lipopolysaccharide Gel Staining Kit (Molecular Probes) following the manufacture's instruction. Silver staining was also performed on the gels that had been previously stained with ProQ300Emerald using the Silver Stain Plus Kit (BIORAD), following the manufacture's instruction. Pictures of gels were taken with ChemiDoc™ MP Imaging System (BIORAD), and the images were analyzed by Image Lab 6.0 Software (BIORAD).

Semi-quantification of short-O-antigen LPS molecules (*continued from the Main Text*). Band intensities were measured from ProQ300Emerald stained gels using Image Lab software. Each band was normalized to the corresponding band for the wild type, per growth condition (log or stationary phase). The data were graphed and statistically analyzed using Prism 8 software (GraphPad Software, La Jolla, CA, USA).

REFERENCES

1. McKenzie GJ & Craig NL (2006) Fast, easy and efficient: site-specific insertion of transgenes into enterobacterial chromosomes using Tn7 without need for selection of the insertion event. *Bmc Microbiol* 6:39.
2. Topp S*, et al.* (2010) Synthetic Riboswitches That Induce Gene Expression in Diverse Bacterial Species. *Appl Environ Microb* 76(23):7881-7884.
3. Gibson DG*, et al.* (2009) Enzymatic assembly of DNA molecules up to several hundred kilobases. *Nat Methods* 6(5):343-345.
4. Datsenko KA & Wanner BL (2000) One-step inactivation of chromosomal genes in Escherichia coli K-12 using PCR products. *Proc Natl Acad Sci U S A* 97(12):6640-6645.
5. Castanie-Cornet MP, Cam K, & Jacq A (2006) RcsF is an outer membrane lipoprotein involved in the RcsCDB phosphorelay signaling pathway in Escherichia coli. *J Bacteriol* 188(12):4264-4270.
6. Bligh EG & Dyer WJ (1959) A rapid method of total lipid extraction and purification. *Canadian journal of biochemistry and physiology* 37(8):911-917.
7. Dalebroux ZD*, et al.* (2015) Delivery of cardiolipins to the Salmonella outer membrane is necessary for survival within host tissues and virulence. *Cell Host Microbe* 17(4):441-451.
