# Supplementary material for: Salmonella enterica Serovar Typhimurium Uses PbgA/YejM To Regulate Lipopolysaccharide Assembly during Bacteremia
Source: Infect Immun. 2019 Dec 17;88(1):e00758-19. doi: 10.1128/IAI.00758-19 (PMC6921655; doi:10.1128/IAI.00758-19)
Supplement: Supplemental file 2 [file IAI.00758-19-s0002.pdf]

Table S1. Bacterial strains and plasmids used in this study

Strain or plasmid	Genotype and notes	Reference
***Salmonella enterica* serovar Typhimurium 14028s**		
ZD004	Wild type (*pbgA+*) *wza-lacZ*	(1)
ZD002	*pbgAΔ328-586::tetRA wza-lacZ*	(2)
ZD003	*pbgAΔ191-586::tetRA wza-lacZ*	(2)
ZD015	*pbgAΔ191-586::tetRA att::*Tn7-*pbgA1-586 wza-lacZ*	This study
ZD016	*pbgAΔ191-586::kan wza-lacZ*	This study
ZD017	*pbgAΔ191-586::tetRA yciM-Q112K wza-lacZ*	This study
ZD018	*pbgAΔ191-586::tetRA ftsH-R299C wza-lacZ*	This study
ZD019	*pbgAΔ191-586::tetRA lpxC-Y113C wza-lacZ*	This study
ZD020	*pbgAΔ191-586::tetRA lapB-R273P,R274S wza-lacZ*	This study
ZD021	*pbgAΔ191-586::kan** suppressor isolate *wza-lacZ*	This study
ZD022	*ΔphoPQ::kan wza-lacZ*	This study
ZD023	*ΔphoPQ::kan pbgAΔ191-586::tetRA wza-lacZ-cat*	This study
ZD024	*ΔphoPQ::kan pbgAΔ328-586::tetRA wza-lacZ-cat*	This study
ZD024	*phoQT48I wza-lacZ*	(2)
ZD025	*phoQT48I pbgAΔ191-586::tetRA wza-lacZ-cat*	(2)
ZD026	*phoQT48I pbgAΔ328-586::tetRA wza-lacZ-cat*	(2)
***Salmonella enterica* serovar Typhimurium LT2**		
JSG212	*hsdLT hsdSA hsdSB galE,* rough strain (LB5010)	(3)
Escherichia coli		
DH5-alpha	Transformation and cloning intermediate	ATCC
**Plasmids**		
pKD46	This lambda-red tool encodes *bet, exo,* and *gam* under control of the arabinose inducible PBAD promoter.	(4)
pGRG37	pGRG37 transgene insertion plasmid; Amp^R	(5)
pGRG37-*pbgA*	Encodes full length *pbgA*; Amp^R	This study
pBAV1k	Riboswitch controlled gene expression plasmid, Kan^R	(6)
pBAV1k-*pbgA*	Encodes full length *pbgA*; Kan^R; served as the template for cloning into pGRG37	This study

* This suppressor genome has not been sequenced

Table S2. Primers used in this study

Primer name	Primer sequence (5'→3')
191*pbgAkan*Fwd	TTTTCGTCTCCTTTATCGCATCGCATCTTATCTACATCTGGTGTAGGCT GGAGCTGCTTC
191*pbgAkan*Rvs	AAGGGGAAACGGTGCTAACTGATTTATAATTAAATATGAATATCCTCCT TAGTTCCTATT
*yejL*chkFwd	CCACAACTCTCCCGCTATAG
*kan*chkRvs	GATATTCGGCAAGCAGGC
3'*pbgA*chkRvs	CGATTATTCCCCGTTGCT
Delta*phoPQkan*Fwd	AGCACCATAATCAACGCTAGACTGTTCTTATTGTTAACACGTGTAGGCT GGAGCTGCTTC
Delta*phoPQkan*Rvs	CCGATTATAACGGATGCTTAACGAGATGCGTGGAAGAACGATATGAAT ATCCTCCTTAGTTCCTATTCCGAAG
*phoPQ*chkFwd	CTTCAGAAAGAGGGTGACTATTTGTCTGG
pBAV: Upper_*pbgA*_insert_ATG	GCAGCACCCTGCTAAGGAGGTAACAACAAGATGGTAACTCATCGTCAG CGCTAC
pBAV: Lower_*pbgA*_insert_TGA	GCGGCATCGATCGGGCCCTGAGGCCTGCAGTCAGTTAGCGATAAAAC GTTTCTCTTCTG
pBAV: Upper_T5_ribo_vector	ACAGAAGAGAAACGTTTTATCGCTAACTGACTGCAGGCCTCAGGGC
pBAV: Lower_T5_ribo_vector	TTCACGGTAGCGCTGACGATGAGTTACCATCTTGTTGTTACCTCCTTA GCAGGGT
fwd.PacI_ribo-*pbgA*	AAATTAATTAAATTCGCGGCCGCTTCTAGA
rvs.XhoI_ribo-*pbgA*	AAACTCGAGTCAGTTAGCGATAAAACGTTTCTCTTCTG

Table S3. Membrane phospholipid levels for *S.* Typhimurium.

Level (ng/μl) (avg ± SD) of the individual phospholipid molecules in total membrane fraction assessed by normal-phase liquid chromatography tandem mass spectrometry (LC-MS/MS)

m/z of parent ion (fatty acid monitored as the daughter ion)	Wild type	*pbgAΔ191-586*	*//Tn7*	//Tn7 + 0.5mM Theo	*pbgAΔ191-586 lpxC Y113C*	*pbgAΔ191-586 lapB/yciM R27P,R274S*
			**LOG PHASE**			
acyl-PGI						
955 (C16:1)	0.36 ± 0.18	1.04 ± 0.36	0.41 ± 0.12	1.19 ± 0.31	0.83 ± 0.23	0.98 ± 0.36
958 (C16:0)	1.55 ± 0.71	2.04 ± 0.84	1.54 ± 0.38	2.53 ± 0.39	2.30 ± 0.37	1.98 ± 0.55
986 (C16:0)	1.21 ± 0.44	1.74 ± 0.57	1.5 ± 0.35	1.25 ± 0.13	1.68 ± 0.27	1.19 ± 0.2
1012 (C16:0)	0.49 ± 0.27	0.55 ± 0.3	0.51 ± 0.26	0.74 ± 0.11	0.59 ± 0.07	0.46 ± 0.08
PGI						
719 (C16:1)	61.07 ± 18.79	70.17 ± 15.12	55.24 ± 17.65	77.78 ± 37.6	70.28 ± 15.95	70.12 ± 20.44
733 (cyC17:0)	4.24 ± 0.92	3.07 ± 0.56	3.67 ± 1.66	3.11 ± 2.11	3.16 ± 0.52	3.0 ± 0.86
747 (C18:1)	11.04 ± 2.55	12.71 ± 2.43	8.07 ± 2.42	5.65 ± 2.98	7.19 ± 1.17	6.53 ± 1.78
759 (cyC17:0)	5.76 ± 0.89	3.10 ± 0.45	3.14 ± 1.01	4.95 ± 2.43	4.14 ± 0.44	3.77 ± 1.1
773 (C18:1)	30.06 ± 11.68	26.23 ± 6.8	20.29 ±4.11	29.98 ± 14.74	24.56 ± 7.32	22.58 ± 7.6
CL*						
1322 (C16:1)	4.37 ± 2.28*	2.7 ± 1.42*	1.92 ± 0.83*	1.17 ± 0.67*	2.1 ± 2.09*	1.65 ± 0.44*
1348 (C16:1)	48.55 ± 18.65*	48.49 ± 20.57*	31.11 ± 12.42*	36.57 ± 16.26*	38.03 ± 6.29*	35.38 ± 5.21*
1376 (C18:1)	31.01 ± 8.83*	36.22 ± 7.8*	24.66 ± 7.27*	19.44 ± 6.06*	25.08 ± 5.87*	23.68 ± 6.33*
1402 (C18:1)	18.44 ± 5.57*	20.60 ± 6.35*	8.60 ± 2.20*	6.60 ± 2.39*	6.79 ± 1.28*	5.24 ± 1.17*
PE						
688 (C16:1)	27.95 ± 12.42	25.29 ± 8.04	39.22 ± 10.35	56.09 ± 18.60	47.55 ± 8.61	45.26 ± 12.04
702 (cyC17:0)	7.95 ± 2.46	5.73 ± 1.30	13.22 ± 5.16	9.64 ± 4.43	9.99 ± 1.68	7.39 ± 1.59
714 (C16:1)	12.40 ± 5.06	10.04 ± 3.87	14.93 ± 4.38	41.10 ± 15.51	29 ± 4.7	24.61 ± 9.28
716 (C18:1)	36.18 ± 9.62	55.14 ± 11.53	52.21 ± 5.29	40.18 ± 6.92	48.66 ± 2.66	42.95 ± 7.18
742 (C18:1)	48.53 ± 20.21	46.76 ± 8.97	46.41 ± 5.72	76.47 ± 28.26	66.01 ± 10.07	60.73 ± 25.80
			**STATIONARY PHASE**			
acyl-PGI						
955 (C16:1)	0.007 ± 0.001	0.013 ± 0.014	0.015 ± 0.006	0.013 ± 0.004	0.007 ± 0.001	0.009 ± 0.002
958 (C16:0)	0.047 ± 0.017	0.076 ± 0.063	0.098 ± 0.028	0.060 ± 0.019	0.060 ± 0.010	0.054 ± 0.014
986 (C16:0)	0.045 ± 0.021	0.109 ± 0.106	0.084 ± 0.020	0.032 ± 0.014	0.064 ± 0.021	0.047 ± 0.013
1012 (C16:0)	0.022 ± 0.014	0.023 ± 0.014	0.027 ± 0.014	0.025 ± 0.012	0.028 ± 0.011	0.032 ± 0.017
PGI						
719 (C16:1)	1.3 ± 0.61	2.0 ± 1.26	3.26 ± 0.78	2.01 ± 0.57	1.25 ± 0.35	1.96 ± 0.51
733 (cyC17:0)	**2.38 ± 0.5**	**0.87 ± 0.42**	**2.87 ± 0.35**	**2.64 ± 0.65**	**2.68 ± 0.57**	**3.7 ± 0.43**
747 (C18:1)	0.22 ± 0.06	0.44 ± 0.26	0.57 ± 0.07	0.21 ± 0.06	0.31 ± 0.06	0.35 ± 0.09
759 (cyC17:0)	0.69 ± 0.31	0.98 ± 0.96	1.44 ± 0.28	1.71 ± 0.39	1.49 ± 0.39	1.95 ± 0.56
773 (C18:1)	0.47 ± 0.29	0.63 ± 0.51	1.95 ± 0.48	1.23 ± 0.33	0.99 ± 0.26	1.18 ± 0.34
CL*						
1322 (C16:1)	0.09 ± 0.04*	0.13 ± 0.06*	0.69 ± 0.16*	0.25 ± 0.09*	0.20 ± 0.05*	0.25 ± 0.08*
1348 (C16:1)	0.92 ±0.49*	1.98 ± 0.93*	5.26 ± 1.96*	2.24 ± 0.80*	2.28 ± 0.81*	2.84 ± 0.44*
1376 (C18:1)	0.76 ± 0.34*	2.09 ±0.82*	4.57 ± 1.07*	1.32 ± 0.44*	2.21 ± 0.56*	1.92 ± 0.26*
1402 (C18:1)	0.45 ± 0.19*	1.77 ± 0.56*	2.52 ± 0.57*	0.68 ± 0.25*	1.46 ± 0.36*	1.13 ± 0.20*
PE						
688 (C16:1)	0.09 ± 0.04	0.38 ± 0.37	0.28 ± 0.1	0.15 ± 0.04	0.07 ± 0.02	0.13 ± 0.04
702 (cyC17:0)	**5.68 ± 0.79**	**2.87 ± 0.6**	**6.29 ± 1.09**	**5.93 ± 0.92**	**5.32 ± 0.63**	**6.76 ± 0.61**
714 (C16:1)	0.04 ± 0.01	0.17 ± 0.12	0.17 ±0.08	0.15 ± 0.04	0.06 ± 0.01	0.13 ± 0.05
716 (C18:1)	0.94 ± 0.27	3.92 ± 1.62	2.86 ± 0.75	0.66 ± 0.14	1.09 ± 0.21	1.05 ± 0.3
742 (C18:1)	0.69 ± 0.36	4.16 ± 1.63	2.83 ± 0.73	1.83 ± 0.47	1.44 ± 0.38	2.0 ± 0.67

Phospholipids were extracted from the membranes of log and stationary phase cultures of the wild type, the *pbgA* mutant, the complementation genotype (//Tn7 and //Tn7 + 0.5mM Theo), and the suppressors (*pbgA*$^{\Delta 191\text{-}586}$ *lpxC* ^{Y113C} and *pbgA*$^{\Delta 191\text{-}586}$ *lapB* $^{R273P, R274S}$). Concentrations were measured using curves from commercial standards. Shown is the average (ng/μl) ± SD from at least eight biological replicates per growth condition. CL, cardiolipin; PGI, phosphatidylglycerol; acyl-PGI, acyl-phosphatidylglycerol; PE, phosphatidylethanolamine; cy, cyclopropane. C, number of carbons atoms in a given acyl side chain. The number following the

colon indicates number or unsaturations within acyl chain. (*) Note: Due to the chemical nature of CL, doubly charged anions representing one half of the CL molecules were monitored as parent ions. The levels of CL can be compared between the genotypes and growth conditions. However, the relative abundance of CL compared to the other phospholipids within or between samples cannot be calculated from these data.

Table S4. Outer Membrane glycerophospholipid (GPL) levels for *S.* Typhimurium.

Level (ng/µl) (avg ± SD) of the individual GPL molecules in total membrane fraction assessed by normal-phase liquid chromatography tandem mass spectrometry (LC-MS/MS)

m/z of GPL parent ion (daughter ion monitored)	Wild type	*pbgAΔ191-586*	//Tn7	//Tn7 + 0.5mM Theo	*pbgAΔ191-586 lpxC Y113C*	*pbgAΔ191-586 lapB/yciM R27P,R274S*
	**STATIONARY PHASE**					
acyl-PGI						
955 (C16:1)	0.010 ± 0.002	0.025 ± 0.014	0.021 ± 0.007	0.017 ± 0.003	0.019 ± 0.008	0.025 ± 0.008
958 (C16:0)	0.189 ± 0.065	0.220 ± 0.128	0.374 ± 0.061	0.233 ± 0.040	0.304 ± 0.091	0.384 ± 0.104
986 (C16:0)	0.232 ± 0.048	0.314 ± 0.169	0.392 ± 0.030	0.159 ± 0.025	0.368 ± 0.090	0.411 ± 0.067
1012 (C16:0)	0.115 ±0.050	0.064 ± 0.052	0.165 ± 0.054	0.152 ± 0.055	0.222 ± 0.081	0.368 ± 0.096
PGI						
719 (C16:1)	1.190 ± 0.850	2.406 ± 1.101	2.560 ± 0.734	1.540 ± 0.469	1.478 ± 0.586	2.113 ± 1.168
733 (cyC17:0)	2.357 ± 0.645	1.328 ± 0.396	2.721 ± 0.488	2.596 ± 0.861	4.035 ± 0.888	4.845 ± 1.185
747 (C18:1)	0.234 ± 0.135	0.696 ± 0.250	0.494 ± 0.112	0.215 ± 0.072	0.400 ± 0.129	0.403 ± 0.190
759 (cyC17:0)	0.222 ± 0.079	0.412 ± 0.293	0.398 ± 0.061	0.628 ± 0.167	0.618 ± 0.246	0.639 ± 0.224
773 (C18:1)	0.490 ± 0.421	0.911 ± 0.535	1.027 ± 0.144	1.036 ± 0.345	0.947 ± 0.297	0.954 ± 0.266
CL*						
1322 (C16:1)	1.783 ± 0.578	0.941 ± 0.364	3.012 ± 1.294	0.859 ± 0.193	0.937 ± 0.321	1.252 ± 0.414
1348 (C16:1)	9.902 ± 2.983	10.138 ± 3.467	18.265 ± 5.526	6.517 ± 1.116	6.125 ± 3.345	7.408 ± 1.456
1376 (C18:1)	8.138 ± 1.709	13.003 ± 3352	13.207 ± 3.149	3.497 ± 0.830	6.019 ± 2.540	5.808 ± 1.272
1402 (C18:1)	5.576 ± 0.912	16.398 ± 4.785	11.073 ±2.835	2.233 ± 0.767	5.073 ± 1.853	4.028 ± 0.644
PE						
688 (C16:1)	0.061 ± 0.025	0.601 ± 0.320	0.271 ± 0.097	0.148 ± 0.049	0.086 ± 0.041	0.114 ± 0.030
702 (cyC17:0)	5.636 ± 1.406	5.626 ± 1.594	9.146 ± 1.985	7.936 ± 2.438	8.290 ± 2.063	9.868 ± 1.876
714 (C16:1)	0.104 ± 0.024	0.411 ± 0.180	0.370 ± 0.090	0.360 ± 0.060	0.230 ± 0.100	0.380 ± 0.180
716 (C18:1)	1.147 ± 0.370	9.491 ± 3.790	4.324 ± 1.160	0.898 ± 0.110	1.784 ± 0.650	1.923 ± 0.660
742 (C18:1)	2.051 ± 0.789	11.054 ± 4.003	7.639 ± 1.184	4.540 ± 1.417	4.556 ± 1.840	6.050 ± 2.387

Glycerophospholipids (GPL) were extracted from the outer membranes of log and stationary phase cultures of the wild type, the *pbgA* mutant, the complementation genotype (//Tn7 and //Tn7 + 0.5mM Theo), and the suppressors (*pbgA*$^{\Delta 191\text{-}586}$*lpxC*^{Y113C} and *pbgA*$^{\Delta 191\text{-}586}$*lapB/yciM/*^{R273P,R274S}). Concentrations were measured using curves from commercial standards. Shown is the average (ng/µl) ± SD from at least eight biological replicates per growth condition. CL, cardiolipin; PGI, phosphatidylglycerol; acyl-PGI, acyl-phosphatidylglycerol; PE, phosphatidylethanolamine; cy, cyclopropane. C, number of carbons atoms in a given acyl side chain. The number following the colon indicates number or unsaturations within acyl chain. (*****) Note: Due to the chemical nature of CL, doubly charged anions representing one half of the CL molecules were monitored as parent ions. The levels of CL can be compared between the genotypes and growth conditions. However, the relative abundance of CL compared to the other GPLs within or between samples cannot be calculated from these data.

REFERENCES

1. C. Farris, S. Sanowar, M. W. Bader, R. Pfuetzner, S. I. Miller, Antimicrobial peptides activate the Rcs regulon through the outer membrane lipoprotein RcsF. *J Bacteriol* **192**, 4894-4903 (2010).
2. Z. D. Dalebroux *et al.*, Delivery of cardiolipins to the Salmonella outer membrane is necessary for survival within host tissues and virulence. *Cell Host Microbe* **17**, 441-451 (2015).
3. A. M. Prouty, J. C. Van Velkinburgh, J. S. Gunn, Salmonella enterica serovar typhimurium resistance to bile: identification and characterization of the tolQRA cluster. *J Bacteriol* **184**, 1270-1276 (2002).
4. K. A. Datsenko, B. L. Wanner, One-step inactivation of chromosomal genes in Escherichia coli K-12 using PCR products. *Proc Natl Acad Sci U S A* **97**, 6640-6645 (2000).
5. G. J. McKenzie, N. L. Craig, Fast, easy and efficient: site-specific insertion of transgenes into enterobacterial chromosomes using Tn7 without need for selection of the insertion event. *Bmc Microbiol* **6**, 39 (2006).
6. S. Topp *et al.*, Synthetic riboswitches that induce gene expression in diverse bacterial species. *Appl Environ Microbiol* **76**, 7881-7884 (2010).
