# Supplementary material for: Salmonella enterica Serovar Typhimurium Uses PbgA/YejM To Regulate Lipopolysaccharide Assembly during Bacteremia
Source: Infect Immun. 2019 Dec 17;88(1):e00758-19. doi: 10.1128/IAI.00758-19 (PMC6921655; doi:10.1128/IAI.00758-19)
Supplement: Supplemental file 3 [file IAI.00758-19-s0003.pdf]

SUPPORTING FIGURES AND LEGENDS:

Fig S1.

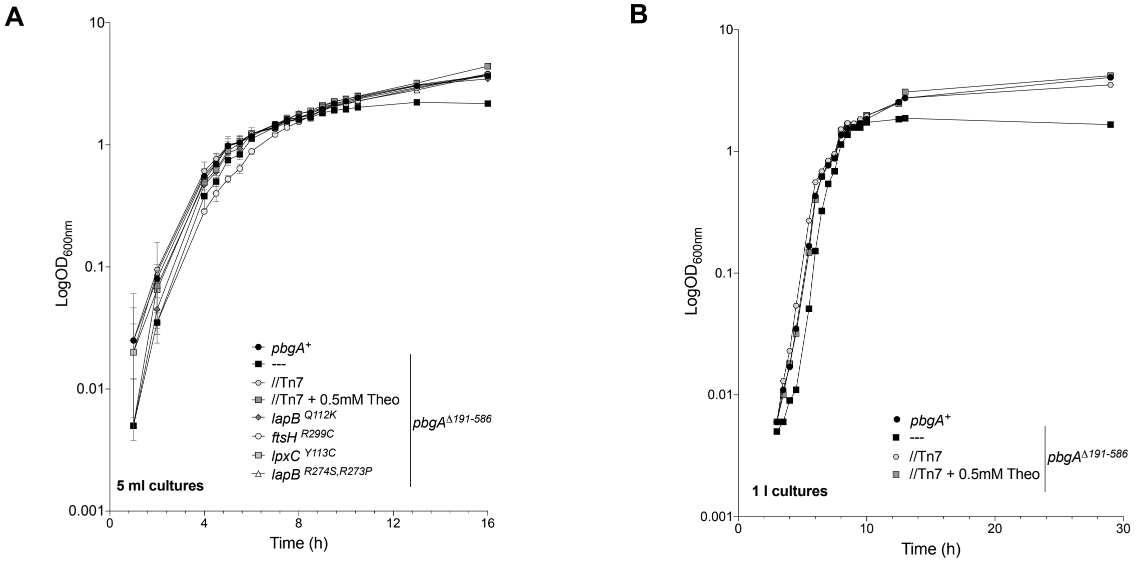

Fig S1. *Salmonella enterica* serovar Typhimurium (*S*. Typhimurium) *pbgA* mutants are attenuated at the transition from log to stationary phase, and complementation and suppression repair the defect. (Related to Fig 2A). Single bacterial colonies were inoculated into **A.** 5ml, or **B.** 1L of Luria-Bertani (LB) broth medium and cultured with continuous agitation at 37ºC for 16h (**A**) and 30h (**B**). The data reflect the mean ± Standard Deviation (SD) of duplicate cultures and representative of two independent experiments for which the OD_{600nm} measurements were acquired at 0.5-1.0h intervals.

Fig S2.

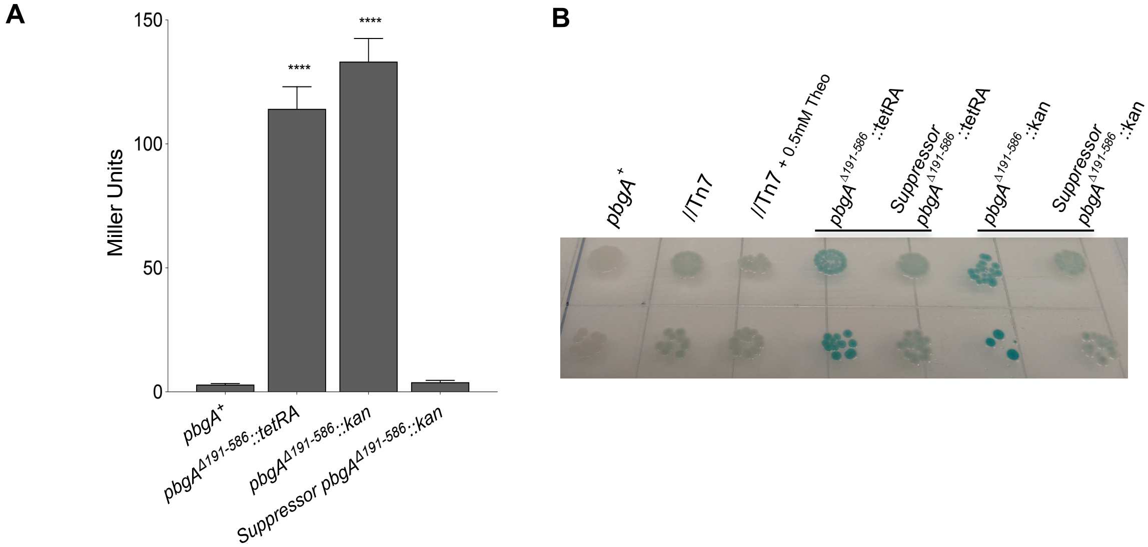

Fig. S2. The *pbgA*-mutant phenotypes are independent of the resistance cassette and kanamycin (km)-resistant genotypes evolve suppressors that are repaired for OM integrity and intracellular survival in macrophages. Beta-galactosidase activity was measured in stationary phase cultures of strains containing a chromosomally integrated *wza-lacZ* gene reporter. The *pbgAΔ191-586*::*km*- suppressor variant emerged from broth culture, and the genome has not been sequenced. Average values (Miller Units) ± Standard Error (SEM) were calculated. Results are representative of at least three independent experiments. One-way ANOVA followed by Bonferroni test was done. **B.** Each strain was cultured to stationary phase in LB broth, diluted, normalized by OD_{600nm} and plated onto LB agar with X-Gal (20µg/ml).

Fig S3.

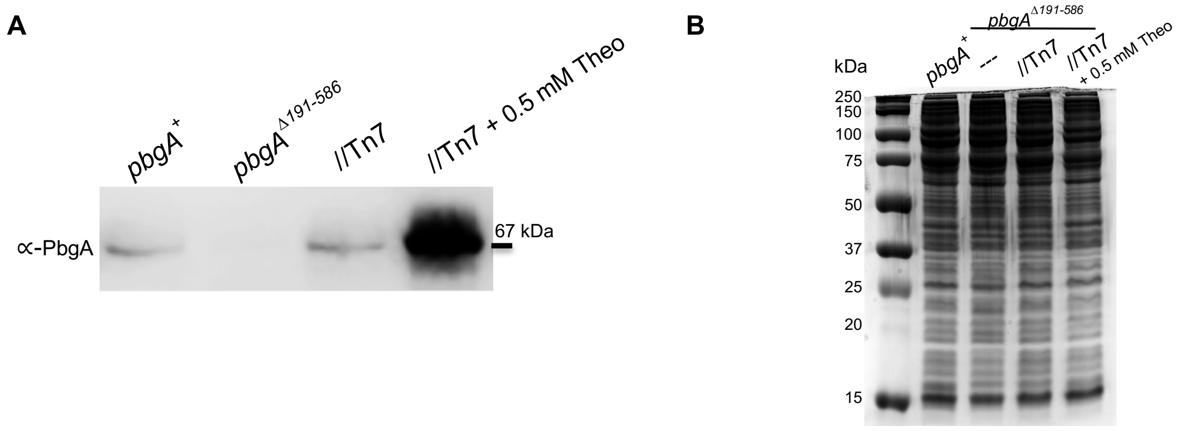

Fig. S3. Adding theophylline (Theo) to the *pbgA*-deletion mutants, which harbor the chromosomally integrated second-site copy of *pbgA*, increases the levels of PbgA expression. A. Shown is a Western blot of the total membrane fractions from the wild type, the *pbgA* mutant, and the complementation genotype (*att::Tn7-pbgA*+) cultured to stationary phase with or without theophylline, (*see Materials and Methods*). The blot is representative of at least three independent experiments**.**

B. Shown is a coomassie-blue stained gel of the proteins in the total membrane fractions of the indicated strains. The gel was loaded and electrophoresed alongside the gels used for the blots represented in **A**.

Fig S4.

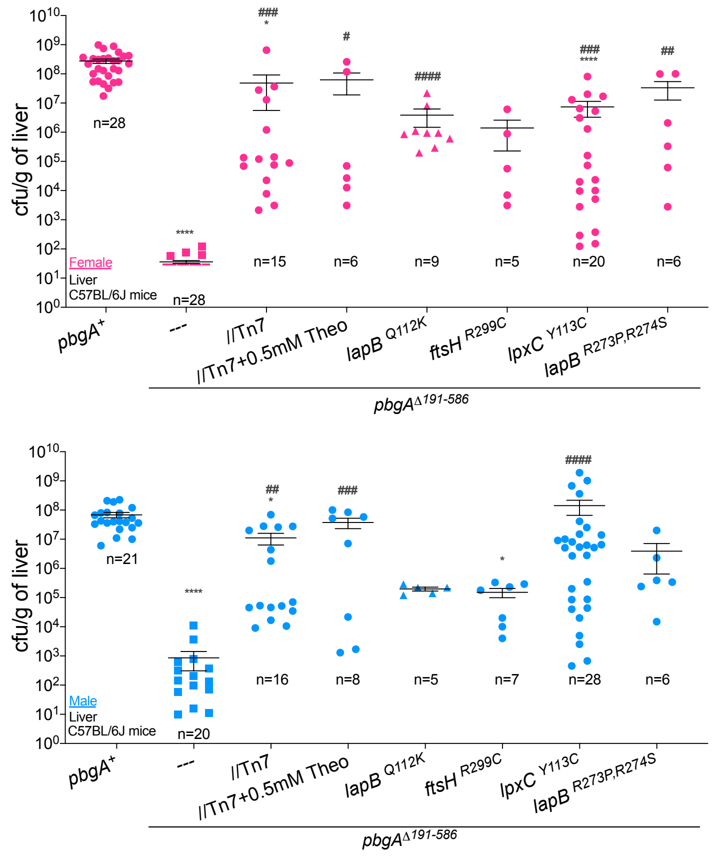

Fig. S4. *S.* Typhimurium require the periplasmic domain of PbgA to colonize the livers of mice following intraperitoneal injection, and *pbgA*-mutant suppressor variants partially restore colonization (Related to Fig. 3A). Female (upper panels) and male (lower panels) C57BL/6J mice were intraperitoneally (i.p.) injected with ~5x10^5 wild type, *pbgA*-mutant, complemented mutant, or the *pbgA*-mutant suppressor variants (**Table 1**). After 2 days post-infection (pi), mice were euthanized and colony counts were enumerated from spleen homogenates. Data shown as mean colony forming units (cfu)/g of liver ± SEM. Each genotype was assessed in at least five mice (n). Some strains were tested in higher number of mice to ensure accuracy. The graph shows data

points at the limit of detection: 30 cfu for livers. A one-way ANOVA and Kruskal-Wallis multi-comparison test was done. Asterisk (*) or number symbol (#) indicates statistically significant relative to wild type or *pbgA*-mutant, respectively.

Fig S5.

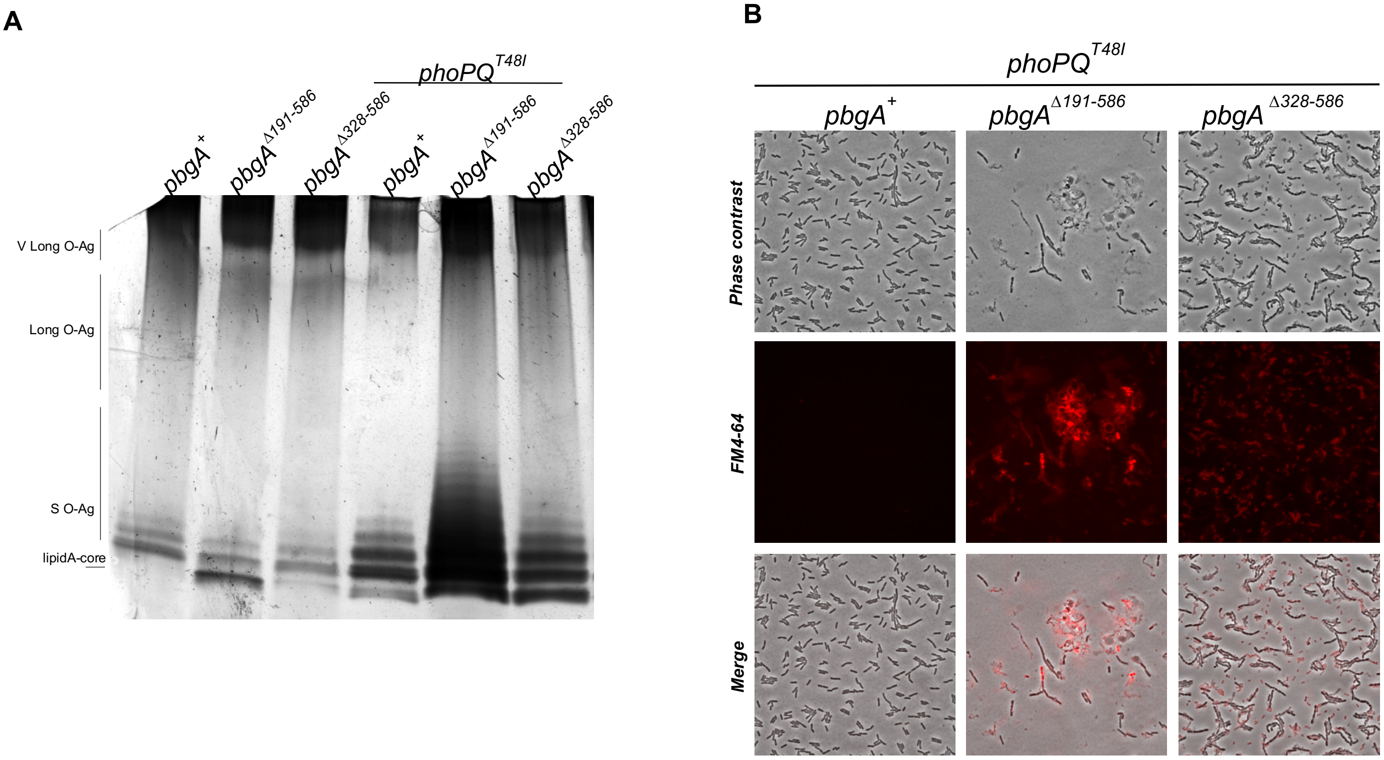

Fig. S5. In the absence of the PbgA periplasmic domain, *S.* Typhimurium that are constitutively activated for PhoPQ signaling accumulate lipidA-core molecules and short-chain LPS molecules and show a severe morphological alteration A. LPS was extracted by hot-phenol method from fresh whole cells, which had been normalized to OD600nm = 2.5. Equivalent amounts of LPS loaded and separated under denaturing conditions using4-20 % gradient gel and visualized with silver stain. Three biological replicates were assessed for each genotype. **B.** Log-phase bacteria were fluorescently labeled with the lipophilic membrane red dye FM4-64 before being spotted onto agarose pads and visualized by phase-contrast and epifluorescence microscopy using 100x magnification.

Fig S6.

A

B

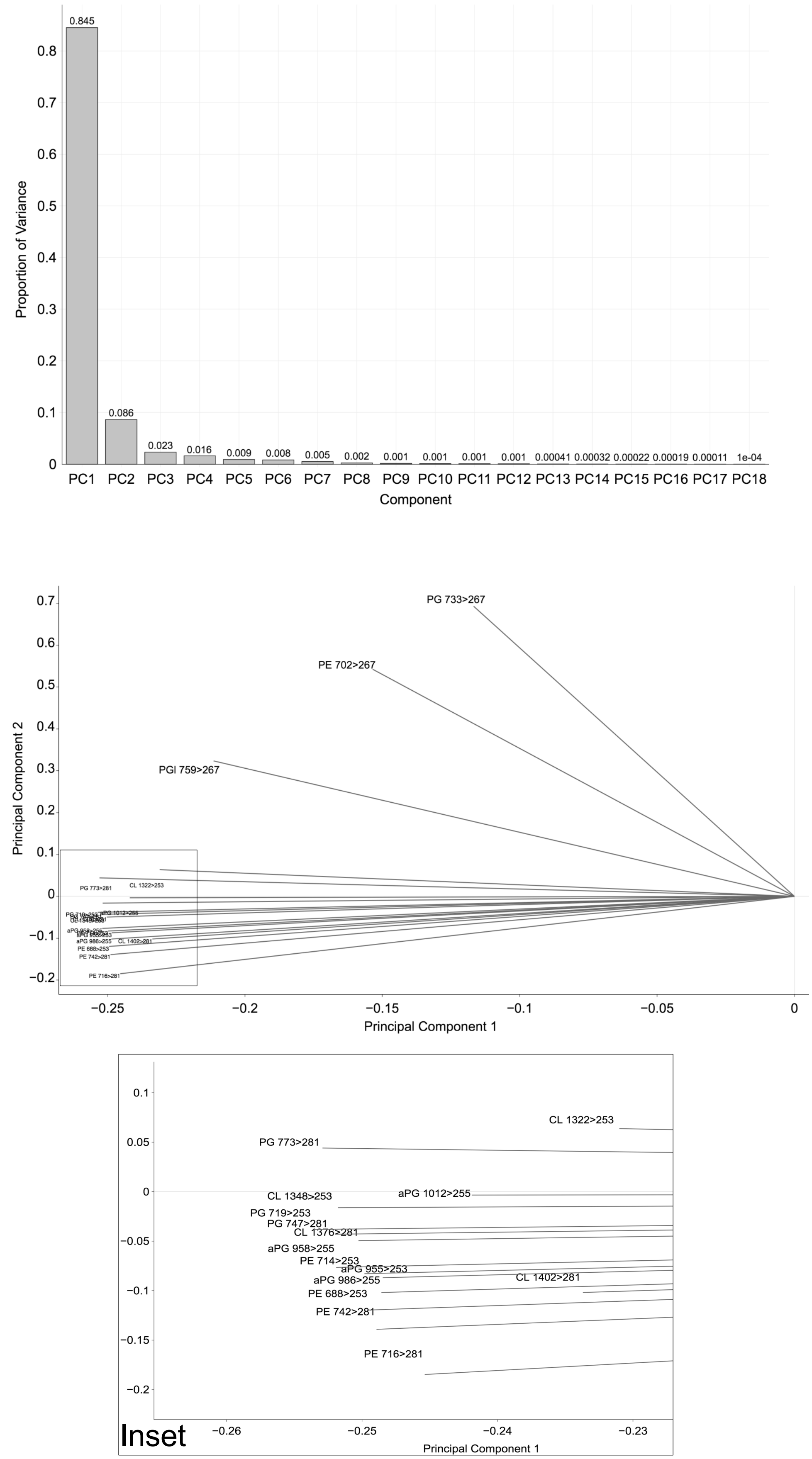

0.845
0.086
0.023
0.016
0.009
0.008
0.005
0.002
0.001
0.001
0.001
0.001
0.00041
0.00032
0.00022
0.00019
0.00011
1e-04
PC1 PC2 PC3 PC4 PC5 PC6 PC7 PC8 PC9 PC10 PC11 PC12 PC13 PC14 PC15 PC16 PC17 PC18
Proportion of Variance
Component
PG 733>267
PE 702>267
PGI 759>267
Principal Component 2
Principal Component 1
CL 1322>253
PG 773>281
CL 1348>253
aPG 1012>255
PG 719>253
PG 747>281
CL 1376>281
aPG 958>255
PE 714>253
aPG 955>253
aPG 986>255
CL 1402>281
PE 688>253
PE 742>281
PE 716>281
Inset

Fig. S6. Multivariate statistical analysis of the phospholipid concentrations in the total membrane fractions of the *S.* Typhimurium strains used in our study for both the log and the stationary phase of growth. A. (**Related to Fig. 7A)**. Depicted is a Scree plot, which indicates the proportion of variance explained by each principal component in the principal component analysis (PCA). The first two components cumulatively explained 93.1% of the total phospholipid variation. **B.** Shown is a PCA loading plot, which determines the phospholipids with the greatest contribution to the variation for PC1 and PC2. The inset shows a magnification of the loading plot area.

Fig S7.

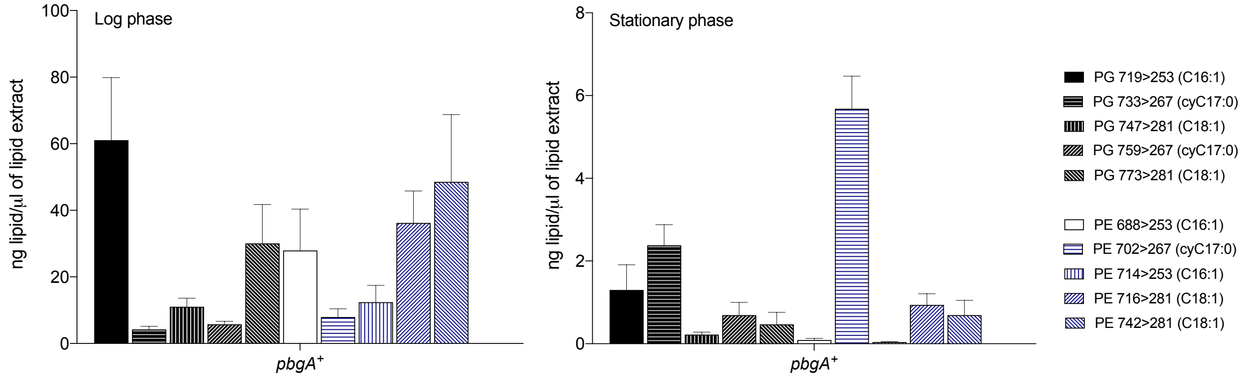

Fig S7. In response to stationary phase stress, *S.* Typhimurium increase the levels of phospholipids with cyclopropanated fatty acids in their membranes. Liquid chromatography tandem mass spectrometry (LC-MS/MS) was performed and standard curves were generated using purified phospholipids in order to quantify the (ng/μl) levels of individual phospholipid species in total membrane fractions. The bar graphs reflect the abundance of individual phospholipid species in the total membrane fractions extracted of the log (left panel) or stationary phase (right panel) *S.* Typhimurium. The phosphatidylglycerols (PGl) and phosphatidylethanolamines (PE) are shown as mean (ng lipid / μl of extract) ± SD between at least eight biological replicates per strain, per growth condition.

Fig S8.

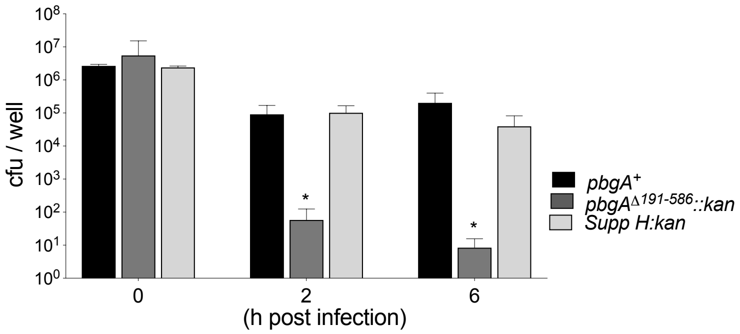

Fig. S8. *S.* Typhimurium *pbgAΔ191-586::km* mutants are attenuated for survival in primary mouse macrophages and un-sequenced suppressor genotypes of this particular *pbgA* mutant restore the intracellular survival defect. (Related to Fig. 8B). Primary bone marrow derived macrophages (BMDM) from C57BL/6J mice were infected with stationary-phase salmonellae at a multiplicity of infection (MOI) of roughly 10:1. The wild type, *pbgA*Δ*191-586*::*km* mutant and the suppressor variant (SuppH::kan) were used to infect triplicate wells of macrophages and the surviving intracellular bacteria were enumerated at two and six hours post infection Three independent experiments were performed. The data are shown as average cfu/well ± SD for each time point. A two-way ANOVA followed by Bonferroni post-test was used to demonstrate statistical differences.

Fig S9.

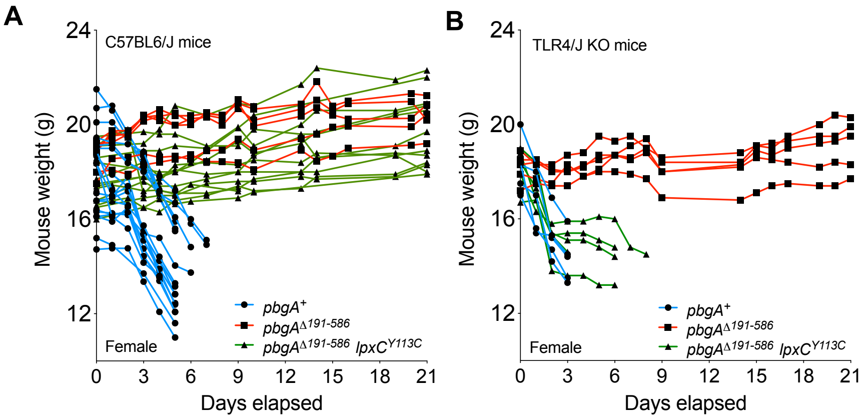

Fig S9. *S.* Typhimurium require the periplasmic domain of PbgA to cause weight loss in mice following intraperitoneal injection, and the *pbgA lpxC* suppressor does not cause weight loss in wild type C57BL/6J mice, but does cause weight loss in Toll-like receptor 4 knockout animals. (Related to Fig. 9). The weight of female of C57BL6/J **(A)** and female C57BL6/J TLR4 KO **(B)** mice infected with wild type (*pbgA*$^{+}$) (●), the *pbgA* mutant (■), or the *pbgA lpxC* suppressor variant (▲) was monitored for 21d or until death. The numbers (n) of mice correlate with the curves shown in **Fig. 9A and D.**

Fig S10.

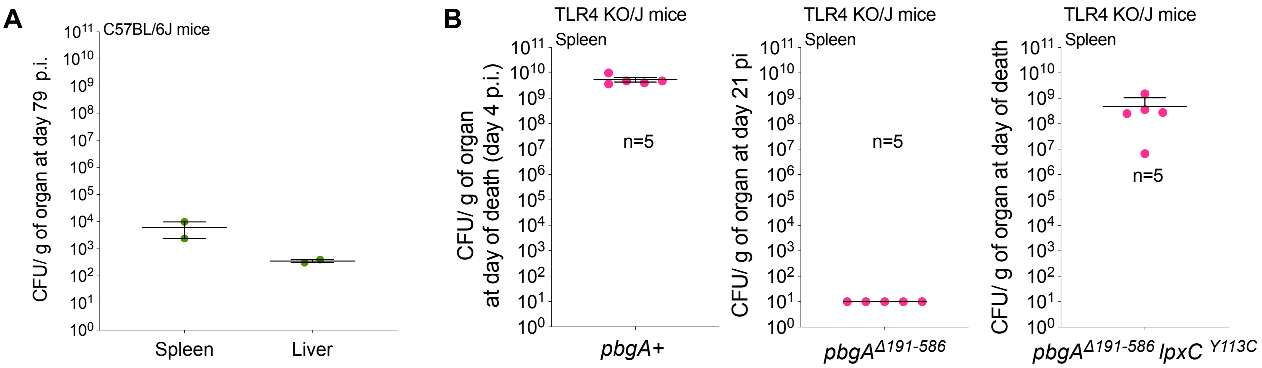

Fig S10. The *pbgA-lpxC* mutant persists at moderate titers in spleens and livers of C57BL/6J mice for months without causing death, but proliferates and causes kills C57BL/6J TLR4 knockout animals (Related to Fig. 9) A. Two C57BL/6J mice that had been infected with the *pbgA-lpxC* salmonellae were sacrificed after seventy nine days and their livers and spleens were dissected and homogenized. **B.** At the time of death (averages: day 4 for *pbgA*+, day 21 for *pbgA* mutant, day 7 for *pbgA-lpxC* suppressor), five C57BL/6J TLR4 mutant mice that had been infected with the genotypes listed were dissected and the spleen homogenates were plated to determine the surviving cfu/g of tissue. For the *wild type* and the *pbgA-lpxC* bacteria, the cfu measurements were made on the day of death. However, since the *pbgA* mutants do not kill the mice, their cfu were quantified at day 21. Our detection limit was 10 cfu per spleen, and no *pbgA* mutant colonies were observed after 21 days in the murine hosts for either the wild type or the knockout animals.
